# Allostery is a widespread cause of loss-of-function variant pathogenicity

**DOI:** 10.1038/s41467-026-74517-8

**Published:** 2026-06-22

**Authors:** Xiaotian Liao, Ben Lehner

**Affiliations:** 1 Wellcome Sanger Institute, Cambridge, UK; 2 University of Cambridge, Cambridge, UK; 3 Centre for Genomic Regulation (CRG), Barcelona Institute for Science and Technology (BIST), Barcelona, Spain; 4 Universitat Pompeu Fabra (UPF), Barcelona, Spain; 5 Institució Catalana de Recerca i Estudis Avançats (ICREA), Barcelona, Spain

**Keywords:** Genetics, Systems biology, Proteomics, Structural biology

## Abstract

Allosteric communication between non-contacting sites in proteins plays a fundamental role in biological regulation and drug action. While allosteric gain-of-function variants are known drivers of oncogene activation, the broader importance of allostery in genetic disease and protein evolution is less clear. Here, we introduce a comparative framework that disentangles functional disruption by mutations from protein destabilization. Applying this framework across diverse datasets—ranging from paired experimental measurements of abundance and activity to proteome-wide comparisons of evolutionary fitness and biophysical stability predictions—we provide evidence that allostery is a widespread cause of loss-of-function variant pathogenicity in human genetic diseases. In addition, our analyses reveal a conserved distance-dependent decay of allosteric mutational effects outside of protein active sites. As an important mechanism of pathogenicity, allostery needs to be better mapped, understood, and predicted across the human proteome.

## Abstract (web summary)

Mutations at sites distant from a protein’s active site can disrupt function through allosteric mechanisms. Here, authors present evidence that allostery is a widespread and underappreciated cause of loss-of-function variant pathogenicity in human diseases.

## Introduction

Allostery, the regulation of a protein’s activity through changes at sites distant from an active site, underpins protein regulation^1–3^. Allostery allows proteins to function as molecular switches and microprocessors, with their enzymatic activities and molecular interactions quantitatively controlled by small-molecule binding, post-translational modifications, and macromolecular interactions. Allostery is central to the control of metabolic pathways, signal transduction, growth control and neural information processing in both normal physiology and disease^1,3–8^. Moreover, many effective therapeutics bind allosteric sites and have allosteric mechanisms of action, including drugs targeting ion channels, receptors, kinases, and transcription factors^2,9–13^.

The importance of allostery for human genetic disease is, however, less well established^5,14,15^. While the role of allostery in gain-of-function (GOF) diseases is well-documented, exemplified by driver mutations in KRAS^16,17^, JAK2^18,19^, and EGFR^20,21^, its contribution to loss-of-function (LOF) human genetic disease remains underexplored^15,22,23^. While it has been estimated that ~40-60% of LOF variants cause disease by destabilizing the protein fold (reducing abundance)^24–28^, a substantial fraction retain stability but fail to function^27,29–37^. These variants likely disrupt specific functional sites - not only orthosteric active sites but also distal allosteric positions, which form the dynamic networks and energetic pathways essential for signal propagation through the protein structure. We term these variants ‘functional mutations’ to distinguish them from variants affecting protein stability.

The potential scale of such distal functional disruption is underscored by recent comprehensive mutagenesis studies, which have generated the first systematic experimental maps of allosteric communication throughout proteins^35–39^. These near-complete atlases for a handful of proteins have revealed that many mutations—even those distant from active sites and binding sites—can exert measurable allosteric effects on protein function. For example, 114 amino acid substitutions in the oncoprotein KRAS allosterically inhibit binding to the oncoprotein effector RAF1^37^. Even in small protein domains such as SH3 domains, many variants have small but measurable allosteric effects^35^ that, in combination, can strongly impair binding to interaction partners^40^. These first allosteric maps suggest that allostery is widespread and highlight the need to reconsider the importance of allostery for variant pathogenicity and molecular evolution.

Predicting these energetic networks computationally has traditionally relied on identifying co-evolution^41–44^. Because allosteric communication requires energetic coupling between distant residues, a mutation at one site creates evolutionary pressure for compensatory mutations at coupled sites. Conventional statistical methods, such as Statistical Coupling Analysis (SCA), and Direct Coupling Analysis (DCA), exploited this signal to map “sectors” and pairs of co-evolving residues that correspond to functional allosteric networks^45–48^.

Recent advances in deep learning have scaled this logic to the entire proteome^49–51^. Protein Language Models (pLMs) like ESM-1v^49^ are trained on millions of sequences and learn to predict the probability of an amino acid given its context. Crucially, these models implicitly learn “total evolutionary fitness”, capturing not just folding constraints, but also potentially the long-range statistical dependencies (allostery) and binding interactions important for survival. However, pLMs do not distinguish the mechanism by which a variant is deleterious. To disentangle these mechanisms, we require models such as ThermoMPNN^52^, which are trained on biophysical data and predict the folding free energies of mutations.

Here, we integrate these complementary computational models to identify pathogenic variants that act through functional disruption rather than stability loss. We first validate this framework using clinically important proteins (PTEN, GCK) where paired experimental measures of abundance and activity allow us to distinguish variants that impair molecular function from those that reduce protein abundance. We then extend this analysis to essential DNA repair proteins (BRCA1, RAD51C, BAP1) profiled by saturation genome editing, demonstrating that predicted stability helps isolate functional mutations. Next, we turn to comprehensive experimental allosteric maps of binding domains (PSD-95-PDZ3, GRB2-SH3) to confirm that the residual variance between predicted ESM-1v fitness and measured folding stability captures changes in binding free energy. Finally, we scale this analysis to six diverse human enzymes to demonstrate the universality of these observations. Across multiple analyses and structurally diverse proteins, these LOF allosteric effects are enriched close to protein active sites and decay over distance. Our results suggest that allostery is a widespread mechanism of variant pathogenicity that needs to be better understood and predicted across the human proteome.

## Results

### Pathogenic variants often act through mechanisms beyond stability

From a structural perspective, missense mutations that impair protein function can act through three primary mechanisms: (1) globally destabilizing the protein fold, (2) directly disrupting the active or binding site, or (3) indirectly perturbing function through distal or allosteric effects. To estimate the relative contribution of stability to LOF pathogenicity, we combined proteome-wide stability predictions, large-scale experimental abundance measurements, and clinical variant annotations.

#### Evaluation of ClinVar variants

**Fig. 1:**
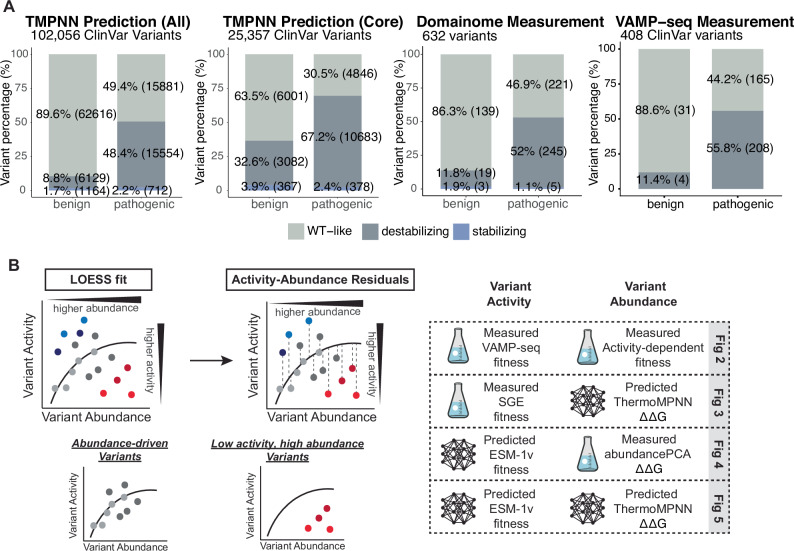
Pathogenic variants often act through mechanisms beyond stability. **A** Classification of ClinVar missense variants into three categories: WT-like (light gray), destabilizing (dark gray), and stabilizing (blue), based on their impact on protein stability/abundance. Percentages and total counts are shown for all ClinVar variants included in the proteome analysis (left), protein core variants (middle-left), variants from ‘Domainome 1.0’ (middle-right), and ‘VAMP’-seq (right), stratified by benign and pathogenic clinical labels. TMPNN = ThermoMPNN. **B** Schematic of the “activity-abundance residual” framework used to isolate functional effects. A LOESS regression (black curve) defines the baseline relationship between protein abundance (or predicted stability) and activity (or predicted fitness). Variants tracking the diagonal are “abundance-driven,” where functional loss is commensurate with destabilization. In contrast, “activity-driven” variants (red) deviate significantly from this trend, retaining high abundance/stability but losing function. The vertical distance from the trendline (residual) quantifies this abundance-independent functional effect. The table (right) lists the specific combinations of experimental (flask icon) and computational (network icon) metrics used to calculate these residuals across the different datasets in this study (Figs. 2–5). LOESS Locally Estimated Scatterplot Smoothing. AbundancePCA = Abundance Protein Fragment Complementation Assay.

We first used ThermoMPNN (TMPNN), a stability-prediction model built on the ProteinMPNN^53^ inverse folding network and trained on large-scale experimental data^27^, to predict changes in folding free energy (ΔΔG_f_) for all possible missense mutations across 19,288 human proteins. This analysis encompassed 187,634,329 variants, including 102,056 annotated in the ClinVar database^54,55^. As expected, pathogenic variants were significantly more likely to be predicted as destabilizing than benign variants (48.4% vs 8.8%, odds ratio (OR) = 9.752, *p* < 2.2e-16, Fisher’s exact test (FET)). Predicted stability changes are thus a very useful, albeit imperfect, predictor of pathogenicity (Receiver Operating Characteristic – Area Under the Curve (ROC-AUC) = 0.698, Precision–Recall Area Under the Curve (PR-AUC) = 0.564, *n* = 32,147 pathogenic, *n* = 69,909 benign variants). Nevertheless, a large proportion of pathogenic variants are predicted to be stable. Even when restricting the analysis to the protein core–where mutations are most likely to disrupt folding^56^ (OR = 10.315, *p* < 2.2e-16, FET)–we found that while 67.2% of pathogenic variants are destabilizing, 30.5% of pathogenic variants are predicted to preserve wild-type level stability (Fig. 1A).

We then validated these computational findings using large-scale experimental datasets. We analyzed the ‘Domainome’ and ‘VAMP-seq’ (Variant Abundance by Massively Parallel sequencing) datasets (Supplemental Table 2), which measure cellular protein abundance for mutations throughout hundreds of domains and several full-length proteins. These experimental data closely mirrored the predictions: 52% of pathogenic variants in protein domains and 55.8% of pathogenic variants in full-length proteins were measured to disrupt abundance (Fig. 1A). Consequently, while stability is predictive of pathogenicity, approximately half of all clinically pathogenic missense variants retain near wild-type-level stability. This indicates that a substantial fraction of human genetic disease is driven by mechanisms beyond fold destabilization.

#### Stability explains only a modest fraction of evolutionary fitness

To quantify the extent to which stability accounts for total variant fitness, we compared stability values against ESM-1v, a protein language model that captures “total fitness” based on evolutionary sequence variation^49^.

Across the entire proteome, ThermoMPNN-predicted stability explained only a modest proportion of the variance in ESM-1v scores (Fig. S1C, median R² = 0.224, interquartile range (IQR) 0.125–0.298). Importantly, this result was not specific to ESM-1v; we observed similar results when comparing stability against AlphaMissense (Fig. S1C), confirming that state-of-the-art variant effect predictors capture pathogenic mechanisms distinct from pure thermodynamic stability loss. To ensure this “missing variance” was not simply due to surface-exposed sites, we repeated the analysis for buried residues only (relative solvent accessible surface area (rSASA) < 0.2, excluding disordered and predicted binding-site residues). Even in the protein core, stability still only partially explained predicted pathogenicity (Fig. S1C, median R² = 0.158, IQR 0.096–0.232). Notably, this relationship varied by protein class: stability explained less variance in regulatory proteins such as voltage-gated ion channels (~10%) compared to ~23–26% in ribosomal proteins, immunoglobulins, and T-cell receptors (Fig. S1F).

This observation was further confirmed by experimental data. We compared ESM-1v scores against experimentally measured abundance changes from the ‘Mega-scale’ (protease sensitivity) and ‘Domainome’ (cellular abundance) datasets. Consistently, experimentally quantified abundance changes explained only a modest fraction of the variance in evolutionary fitness (Fig. S1C, median R² = 0.252 and 0.218, respectively). Similarly, for proteins with high-quality VAMP-seq data, abundance changes explained a median of only 26% of the variance in ESM-1v scores. These results demonstrate that protein stability is insufficient to explain evolutionary fitness: a large fraction of variants is deleterious while preserving wild-type stability/abundance.

#### Isolating functional effects via activity-abundance residuals

To systematically distinguish functional defects from abundance-driven effects, we formulated a framework based on “activity-abundance residuals” (Fig. 1B). We modeled the global relationship between protein abundance and activity using LOESS (locally estimated scatterplot smoothing) regression. The trendline defines the baseline expectation: the typical activity retained by a variant given its abundance.

Visualizing variants relative to this LOESS fit reveals two distinct LOF populations. “Abundance-driven variants” track the LOESS diagonal, where loss of activity is commensurate with destabilization. In contrast, “activity-driven variants” deviate significantly from this trend, clustering in the lower-right quadrant where abundance remains high but activity is lost. We reasoned that the residual—the vertical distance between a variant’s observed activity and the LOESS fit—effectively isolates abundance-independent functional effects. Large negative residuals identify variants where loss of function is disproportionate to the loss of abundance, thereby highlighting disrupted active sites, binding interfaces, or long-range allosteric effects.

### Clinical pathogenic variants in PTEN and GCK show allosteric decay

**Fig. 2:**
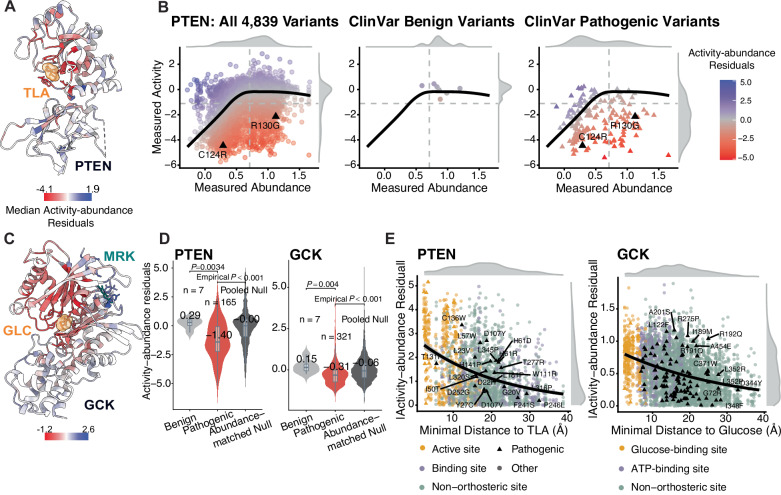
Measured activity vs measured abundance in PTEN and GCK. **A** Crystal structure of PTEN (PDB: 1D5R) colored by the median activity-abundance residuals. L(+)-tartaric acid (TLA), located near the catalytic site, is shown in orange. Regions with large negative residuals (red) indicate sites with high abundance but low activity. **B** Scatter plots of measured protein abundance (‘VAMP’-seq) versus phosphatase activity (yeast complementation) for 4,839 PTEN variants. A LOESS regression curve (black line) models the baseline abundance-activity relationship. Residuals from this fit quantify deviations from the trend. Left: All variants. Center: ClinVar Benign variants (circles). Right: ClinVar Pathogenic variants (triangles). Dashed gray lines indicate wild-type abundance (0.71) and activity (−1.11) values. Two canonical pathogenic mutations at the active site, C124R and R130G, are highlighted. **C** Crystal structure of GCK (PDB: 1V4S) colored by the median activity-abundance residuals. Glucose (GLC-orange) and the allosteric activator (MRK-dark cyan) are highlighted. **D** Distributions of activity–abundance residuals for ClinVar benign, pathogenic, and abundance-matched control variants in PTEN (left) and GCK (right). For all distributions, the violins show the kernel density of all values. Within each violin, an embedded boxplot indicates the median (center horizontal line; values labelled) and the interquartile range (IQR), with whiskers extending to 1.5× the IQR; an overlaid vertical line spans the 2.5th–97.5th percentiles (central 95% of values). To generate the abundance-matched null distribution, variants were first binned by their measured protein abundance (0.5-unit window). For each pathogenic variant, a residual score was randomly sampled from the pool of non-pathogenic variants residing in the same abundance bin (1000 bootstrap iterations). Deviation of pathogenic variants from this null expectation was assessed via a one-sided empirical P-value. Comparisons between Benign and Pathogenic variants were performed using a one-sided Wilcoxon rank-sum test. **E** Distance-dependent decay of functional effects. The absolute magnitude of the activity-abundance residual was plotted against the minimal side-chain heavy atom distance to the substrate. An exponential decay model (black curve representing the least-squares fit) was fitted to variants with negative activity-abundance residuals, with 95% bootstrap confidence intervals shown as gray shading. Three-star ClinVar pathogenic variants (variants reviewed by an expert panel) that reside outside of orthosteric sites are marked as black triangles and labeled.

To validate that “activity-abundance residuals” specifically isolate functional and allosteric sites, we analyzed two clinically important proteins where high-quality measurements of both abundance and activity are available: the lipid phosphatase PTEN (Fig. 2A) and the glucose-sensing kinase glucokinase (GCK) (Fig. 2C). PTEN germline variants are associated with a spectrum of disorders including Cowden syndrome and other cancer predisposition syndromes^57^. GCK, on the other hand, acts as the body’s glucose sensor; LOF variants cause Maturity-Onset Diabetes of the Young (GCK-MODY), while GOF variants cause hyper insulinemic hypoglycemia^58–60^.

We applied our residual framework to datasets combining ‘VAMP’-seq (abundance) with yeast-based functional assays for 4,839 PTEN variants^61,62^ and 8,255 GCK variants^33,60^. By fitting a LOESS curve to the abundance-activity landscape, we defined the expected activity for a given stability (Fig. 2B, Fig. S2A). In both proteins, we observed the expected “abundance-driven” population tracking the diagonal. However, a distinct population of variants deviated significantly, retaining high abundance but exhibiting severely compromised function (large negative residuals).

As expected, many pathogenic ClinVar variants reduced protein abundance. However, many others reduced enzymatic activity more than expected given their abundance levels (Fig. 2D). Mapping these residuals onto the protein structures revealed their enrichment in both active sites and distal allosteric regions. In PTEN, high-residual variants localized to the catalytic core and surface-exposed residues, including the WPD, P, and TI loops that form the active site pocket (Fig. 2A). In GCK, pathogenic variants clustered not only in the glucose-binding pocket but also in helix 13, a known allosteric regulator of activity^63,64^ (Fig. 2C). In contrast, clinically benign variants generally maintained wild-type-like abundance and activity and showed minimal residuals (Fig. 2D).

We next asked whether these functional mutational effects follow a physical pattern consistent with allosteric propagation—the transmission of energetic changes from one site in a protein to another, which provides a mechanistic basis for how mutations distant from active sites can still impact function^65,66^. If mutations act solely by locally disrupting active sites, functional effects should be binary (present at the active site, absent elsewhere). However, in the presence of allostery, energetic perturbations should propagate through the scaffold, decaying with distance from the functional site. This distance-dependent allosteric decay is visible in the small number of experimental allosteric maps generated to date^35–39^.

To test this, we plotted the absolute abundance-corrected activity residuals of each variant against its distance from the bound ligand (Fig. 2E). In both PTEN and GCK, we observed a clear decay: variants closest to the orthosteric sites had the strongest effects, and the typical effect size dropped by half over distances of 14.9 Å (PTEN) and 21.2 Å (GCK). This distance-dependent pattern was statistically significant in both proteins. Linear regression of the log-transformed absolute residuals against distance yielded negative slopes (PTEN: slope = –0.034, *P* = 8.78e-08; GCK: slope = –0.045, *P* < 2e-16).

Both PTEN and GCK contain clinically pathogenic variants that clustered in the active site and interface regions—as expected—but many were also located at distal positions (Fig. 2E). To test whether such non-orthosteric pathogenic variants exert functional effects beyond abundance loss, we compared their activity–abundance residuals to those of abundance-matched, non-pathogenic controls. In both orthosteric and non-orthosteric regions, pathogenic variants showed significantly larger residuals than abundance-matched controls (Fig. S2B), indicating greater loss of activity than expected from abundance effects alone. Many pathogenic variants in PTEN and GCK, therefore, likely act through allosteric mechanisms.

### Clinically pathogenic variants in saturation genome editing data

While the analysis of PTEN and GCK shows that many pathogenic variants could act allosterically, paired experimental measurements of abundance and activity are not available for most human proteins. We therefore asked whether this framework could be generalized by substituting measured abundance with predicted thermodynamic stability.

**Fig. 3:**
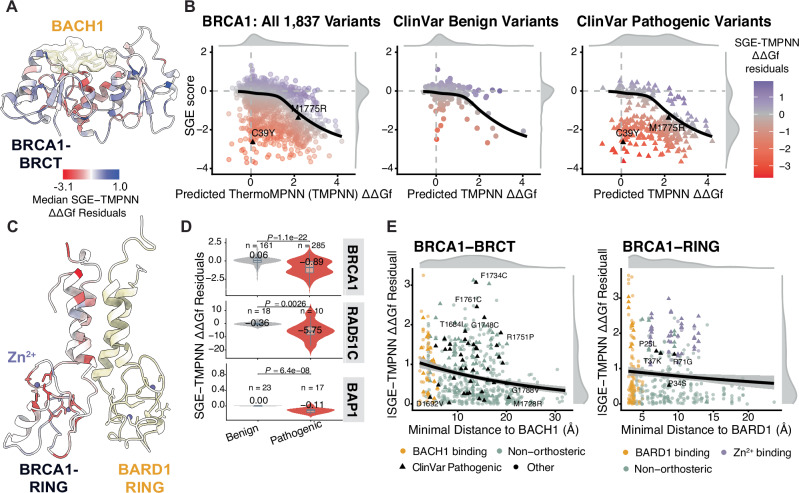
Measured function score vs predicted thermoMPNN stability in SGE proteins. **A** Crystal structure of the BRCA1-BRCT domain (PDB: 1T29). Residues are colored by the median residuals. BACH1 peptide is highlighted in yellow. **B** Scatter plots of experimental function (SGE score) versus predicted ΔΔG_f_ for 1,837 BRCA1 variants. A LOESS regression (black line) defines the baseline relationship. Left: All variants. Center: ClinVar Benign variants (circles). Right: ClinVar Pathogenic variants (triangles). Example pathogenic variants C39Y and M1775R are highlighted. **C** Crystal structure of the BRCA1-RING domain complexed with BARD1-RING domain (yellow) (PDB: 1JM7). **D** Violin plots comparing SGE-ThermoMPNN ΔΔG_f_ residuals between benign and pathogenic variants for BRCA1, RAD51C, and BAP1. For all distributions, the violins show the kernel density of all values. Within each violin, an embedded boxplot indicates the median (center horizontal line; values labelled) and the interquartile range (IQR), with whiskers extending to 1.5× the IQR; an overlaid vertical line spans the 2.5th–97.5th percentiles (central 95% of values). Comparisons between Benign and Pathogenic variants were performed using a one-sided Wilcoxon rank-sum test. **E** Distance-dependent decay of functional residuals. The absolute magnitude of the residual was plotted against the minimal side-chain heavy atom distance to the ligands (BACH1 for BRCA1-BRCT; BARD1-RING for BRCA1-RING). An exponential decay model (black curve representing the least-squares fit) was fitted to variants with negative residuals, with 95% bootstrap confidence intervals shown as gray shading. Three-star ClinVar pathogenic variants (variants reviewed by an expert panel) that reside outside of orthosteric sites are marked as black triangles and labeled.

To test this, we examined three essential DNA repair proteins–BRCA1, RAD51C, and BAP1–for which high-quality functional data are available from Saturation Genome Editing (SGE) screens^67–69^. SGE provides a direct readout of cellular fitness but does not explicitly measure protein abundance. We hence used ThermoMPNN as a computational proxy for structural stability and compared it against the experimental SGE functional scores (Fig. 3, Fig. S3, 4).

Consistent with our previous observations, we found that predicted stability is necessary but insufficient to explain pathogenicity. In BRCA1 (Fig. 3A–C), benign variants were mostly predicted to be stable. However, pathogenic variants split into two distinct populations: those that are severely destabilizing (high ThermoMPNN ΔΔG_f_) and those that are predicted to be highly stable yet are functionally impaired (low SGE score, low ThermoMPNN ΔΔG_f_). Consequently, predicted stability showed only a moderate correlation with experimental function across all three proteins (Fig. S4A, Spearman’s *ρ* = 0.40, 0.40, 0.25, respectively), hinting that a large fraction of pathogenic variants in these proteins likely arise from other mechanisms.

We next calculated the “SGE-ThermoMPNN ΔΔG_f_ residuals” to isolate these functional effects (Fig. 3B, Fig. S3B). In BRCA1, large negative residuals clustered around known functional interfaces. In the BRCT domain, residuals highlighted the phosphorpeptide-binding groove, while in the RING domain, high-residual variants mapped to the zinc ion binding and BARD1 heterodimerization interface (Fig. 3A, C). Quantitatively, pathogenic variants also exhibited significantly larger negative residuals (Fig. 3D).

Importantly, as observed in PTEN and GCK, these functional residuals exhibited the characteristic distance-dependent decay (Fig. 3E, Fig. S3C). In the BRCA1-RING domain, for example, the magnitude of the residuals was the highest at the dimerization interface and zinc ion binding sites and dampened as the distance from the functional site increased.

Finally, we leveraged these high-quality SGE datasets to validate the reliability of our computational models. To determine whether the “missing variance” between predicted stability and fitness reflects genuine biology or model noise, we compared ESM-1v fitness scores directly against the SGE “ground truth” (Fig. S4). To quantify this, we decomposed the variance into the biological signal (ESM-1v–ThermoMPNN ΔΔG_f_) and the predictive noise (SGE –ESM-1v). We found that the biological signal is consistently larger in magnitude than the model noise (Fig. S4B). Further, while the biological signal exhibits a clear distance-dependent decay from orthosteric sites–consistent with physical propagation–the predictive noise is spatially uniform. This shows that the residuals capture a robust, spatially coherent functional signal rather than random model error.

### Experimental allosteric maps confirm distance-dependent decay

**Fig. 4:**
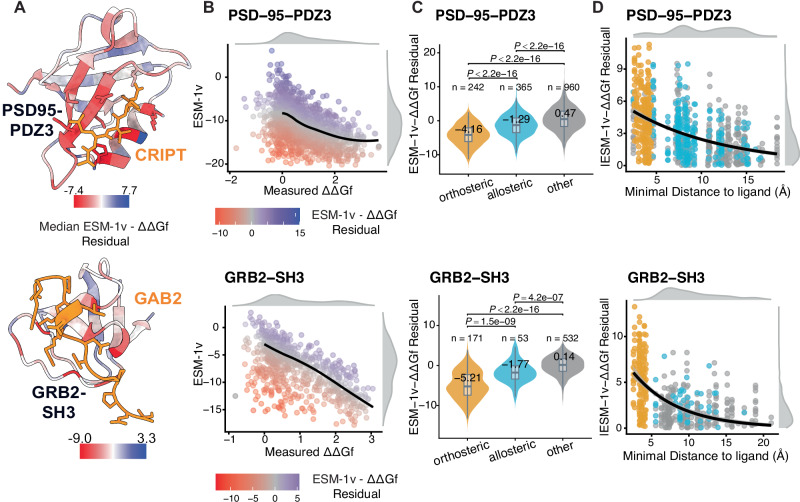
Predicted ESM-1v score vs measured stability in PSD-95-PDZ3 and GRB2-SH3. **A** Crystal structures of PSD-95-PDZ3 bound to CRIPT (A, PDB: 1BE9) and GRB2-SH3 bound to GAB2 (PDB: 2VWF), colored by the median ESM-1v–measured ΔΔG_f_ residual for each site. **B** Scatter plots between predicted ESM-1v and experimentally measured folding stability (top: PSD-95-PDZ3, bottom: GRB2-SH3). A LOESS regression (black line) defines the baseline stability-function relationship. **C** Distributions of ESM-1v–measured ΔΔG_f_ residuals stratified by functional class: orthosteric (direct binding contacts), allosteric (experimentally defined distal functional sites), and others. For all distributions, the violins show the kernel density of all values. Within each violin, an embedded boxplot indicates the median (center horizontal line; values labelled) and the interquartile range (IQR), with whiskers extending to 1.5× the IQR; an overlaid vertical line spans the 2.5th–97.5th percentiles (central 95% of values). Comparisons were performed using a one-sided Wilcoxon rank-sum test. **D** Distance-dependent decay of functional effects. The absolute magnitude of the residual was plotted against the minimal distance to the ligand. An exponential decay model (black curve representing the least-squares fit) was fitted to variants with negative residuals, with 95% bootstrap confidence intervals shown as gray shading. Variants are colored by site type (orange: orthosteric; blue: allosteric; gray: other).

To confirm that these “activity-abundance residuals” specifically isolate binding and allosteric effects, we turned to experimental allosteric maps–datasets where the impact of every mutation on both folding stability (ΔΔG_f_) and binding affinity (ΔΔG_b_) has been explicitly measured. Recent advances have enabled the generation of such comprehensive maps for individual proteins and protein domains^35–39^. In these systems, mutations that alter binding energy without directly contacting the ligand must, by definition, act through an allosteric mechanism. To evaluate this, we focused on two domains with complete mutational maps: the third PDZ domain of PSD-95 and the second SH3 domain of GRB2 (Fig. 4A).

As observed in our proteome-wide analyses, folding stability changes alone explained only a modest fraction of predicted pathogenicity: R² = 0.214 for PSD-95 and R² = 0.359 for GRB2 (Fig. S5A). To isolate the stability-independent functional effects, we fitted a LOESS curve between experimentally measured ∆∆G_f_ and ESM-1v predicted pathogenicity for each domain and computed residuals, quantifying the extent to which mutations disrupt function beyond what would be expected from their impact on folding stability alone (Fig. 4B).

As expected, the largest absolute median residuals were observed at orthosteric binding residues and with experimentally annotated allosteric mutations (Fig. 4C). These effects also followed a spatial pattern consistent with allosteric propagation: absolute residual magnitudes were highest near the binding interface and decayed with increasing 3D distance from the ligand (PSD-95-PDZ3: slope = -0.134, *P* = 6.74e-07; GRB2-SH2: slope = -0.109, *P* = 1.04e-05). Visualizing the ESM-1v–measured ∆∆G_f_ residual further illustrates this distance dependence of predicted functional effects away from the binding interface (Fig. 4D).

We next evaluated experimentally measured ∆∆G_b_ values to directly assess allosteric effects. For residues outside the binding interface, changes in ∆∆G_b_ isolate the allosteric component of binding disruption. These values explained 30.2% of ESM-1v variance in PSD-95 and 4.5% in GRB2 (Fig. S5A). The smaller contribution of ∆∆G_b_ to pathogenicity in GRB2-SH3 likely reflects the domain’s very small size and reduced allostery.

Importantly, binding free energy changes (∆∆G_b_) also showed a distance-dependent decay from the ligand^35–39^ (Fig. S5B). Across residues, ∆∆G_b_ values correlated with ESM-1v–measured ∆∆G_f_ residuals (Fig. S5C), with a stronger relationship in GRB2 (Spearman’s *ρ* = –0.67) than in PSD-95 (ρ = –0.32). Moreover, models incorporating both ∆∆G_f_ and ∆∆G_b_ explained more pathogenicity variance than models using stability alone—even after excluding mutations within the binding interface (Supplemental Tables 4–7). This further demonstrates the allosteric contribution to pathogenicity is independent of changes in fold stability.

### Functional effects decay with distance

**Fig. 5:**
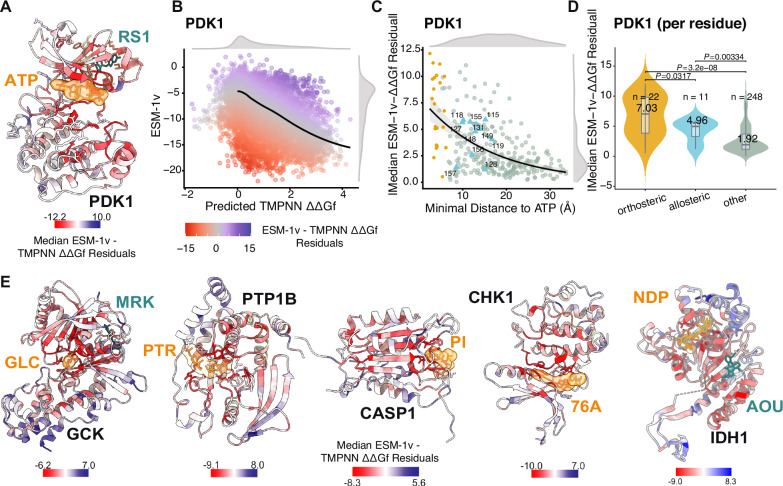
Predicted ESM-1v score vs predicted thermoMPNN stability in allosteric enzymes. **A** Crystal structure of PDK1 (PDB: 4RQK) colored by the median ESM-1v–ThermoMPNN ΔΔG_f_ residuals. The ATP substrate is shown in orange, and the allosteric inhibitor RS1 is labeled in dark cyan. **B** Scatter plot of ThermoMPNN-predicted ΔΔG_f_ versus ESM-1v scores for missense mutations in PDK1. A LOESS curve (black line) models the trend, with residuals quantifying the deviation between predicted ΔΔG_f_ and predicted ESM-1v fitness. **C** Per-residue relationship between the absolute median ESM-1v–ThermoMPNN ΔΔG_f_ residual and the minimal side chain heavy atom distance to the ATP substrate. An exponential decay model (black curve representing the least-squares fit) was fitted, with 95% bootstrap confidence intervals shown as gray shading. Sites are colored by functional class: orthosteric sites (orange), allosteric sites contacting RS1 (cyan triangles), and other sites (sage). **D** Distributions of per-site absolute median residuals for orthosteric, allosteric, and other sites in PDK1. For all distributions, the violins show the kernel density of all values. Within each violin, an embedded boxplot indicates the median (center horizontal line; values labelled) and the interquartile range (IQR), with whiskers extending to 1.5× the IQR; an overlaid vertical line spans the 2.5th–97.5th percentiles (central 95% of values). Comparisons were performed using a one-sided Wilcoxon rank-sum test. **E** Crystal structures of diverse allosteric enzymes colored by median ESM-1v-ThermoMPNN ΔΔG_f_ residuals. Proteins shown: Glucokinase (GCK) (PDB: 1V4S), Protein Tyrosine Phosphatase 1B (PTP1B) (PDB: 1PTY), Caspase-1 (CASP1) (PDB: 2HBQ), Checkpoint Kinase 1 (CHK1) (PDB: 2E9N), and Isocitrate Dehydrogenase 1 (IDH1) (PDB:6IO0). Bound substrates—glucose (GLC), O-phosphotyrosine (PTR), peptide-like inhibitor (PI), CHK1 inhibitor (76A), and NDP—are shown in orange. Known allosteric regulators (MRK, AOU) are highlighted in dark cyan.

The results from experimental data above suggest a general physical principle: deleterious mutations often disrupt function by breaking long-range energetic networks rather than locally destabilizing the fold. To test the universality of this principle, we extended our analysis to a set of six structurally and functionally diverse human allosteric proteins (Fig. 5, Supplemental Table 3). For each allosteric protein in our test set, we compared predicted pathogenicity (ESM-1v) to predicted folding stability changes (ΔΔG_f_) from ThermoMPNN. We then fitted a LOESS curve and computed residuals to quantify variant effects not attributable to stability changes (Fig. 5B, Fig. S6A).

Across all six proteins, many variants exhibited large residuals, indicating functional effects not accounted for by changes in fold stability. ESM-1v and ΔΔG_f_ were moderately correlated (Spearman’s ρ = –0.25 to –0.67), with enrichment of large residuals at annotated orthosteric sites and distal regulatory positions where known allosteric regulators bind (Fig. 5, Fig. S6).

We next asked whether these stability-independent functional effects follow a spatial pattern consistent with allosteric propagation^35–38,65,66^. In all proteins, the absolute median residuals were strongest for residues close to the active site, with the predicted functional effects of mutations decaying with 3D distance from the ligand (Fig. 5C, Fig. S6B). This trend was statistically significant: in a linear model regressing the log-transformed absolute residuals on distance, increasing distance was significantly associated with weaker functional effects (Supplemental Table 3). Across proteins, the half-distances for decay (d_1/2_) are comparable to the distance-dependent decay of allosteric mutational effects observed in experimental allosteric maps^35–39^ (Supplemental Table 3).

We also compared the magnitudes of ESM-1v–ThermoMPNN ΔΔG_f_ residuals at orthosteric sites, distal sites where known allosteric regulators bind, and the rest of the positions. In three out of the six proteins, absolute median residuals were significantly higher at both orthosteric and known allosteric regulator contact sites than at other positions (Fig. 5D, Fig. S6C). In PTP1B, CASP1 and IDH1, allosteric regulator contact sites also showed larger absolute residuals on average (Fig. S6C), although the difference was not statistically significant. It is important to note that this definition of allosteric sites is conservative, as it is based only on the binding sites of known allosteric regulators. Most allosteric proteins will harbor additional allosteric sites that remain untested.

Notably, the distance-dependent decay is not uniform. Some positions exhibit residuals much larger than expected for their distance from the active site. We refer to these as allosteric hotspots—residues with larger functional impact than expected. These hotspots include binding sites of known allosteric regulators, such as inhibitors of protein tyrosine phosphatase 1B (PTP1B) and compound RS1, which targets pyruvate dehydrogenase kinase 1 (PDK1)^70,71^. These findings mirror the distance-dependent decay of mutational effects observed in experimental allosteric maps—including decay from binding interfaces in protein interaction domains^35^, in KRAS^37^, and in activity-altering mutations in the Src kinase^36^, as well as in the allosteric phytohormone receptor PYL1^38^. They are also similar to the decay of abundance corrected mutational effects in SGE genes (Fig. 3) and the two protein binding domains (Fig. 4) presented above.

Finally, benchmarking our computational residuals against diverse experimental datasets^33,35,60–62^ confirms a consistent and significant correlation (Fig. S7). These results suggest that allosteric mechanisms potentially underlie a substantial and underappreciated component of pathogenicity in missense variants, and that the magnitude of these effects decays in a conserved, distance-dependent manner from functional sites.

## Discussion

Taken together, our results indicate that allostery is an important cause of LOF variant pathogenicity. Although many pathogenic variants destabilize proteins^24–28^, changes in stability only partially account for the pathogenicity of variants. Both state-of-the-art computational predictions (Figs. 1,5), and direct experimental measurements (Figs. 2–4) indicate that the indirect, allosteric effects of mutations on activity are a frequent mechanism of pathogenicity. In both PTEN and GCK, for example, many known pathogenic variants have no or weak effects on stability but strong experimentally measured allosteric effects on activity.

To date, comprehensive experimental allosteric maps have only been generated for a handful of proteins^35–39^, and a limitation of our study is the extrapolation of conclusions from these proteins to the whole proteome. Nevertheless, results from our proteome-wide analyses using state-of-the-art computational predictors are highly consistent with the conclusions from the experimental allosteric maps. Ultimately, it will be important in future work to scale up the production of experimental allosteric maps, particularly for proteins that harbor large numbers of clinically observed variants^33,60,62^. Furthermore, we have only experimentally considered protein-protein binding and enzyme catalytic activity in this study; future efforts should develop methods to quantify allosteric effects on additional molecular activities, such as DNA binding and small-molecule interactions^72^. A comprehensive understanding of pathogenic mechanisms will ultimately require experimental tools capable of capturing allosteric regulation across the full spectrum of disease-relevant molecular activities.

Additional caveats should be noted. First, ESM-1v is a proxy for variant pathogenicity and evolutionary fitness derived from protein sequences; while it captures many aspects of mutational impact on protein function, it is not a direct measure of clinical pathogenicity. Consequently, variants with large ESM-1v-∆∆G_f_ residuals that are currently not classified as pathogenic likely represent a combination of unannotated variants, subclinical functional changes that do not manifest as disease, and artifacts of the predictive models. Second, our computational analyses and the equivalent experimental data aim to quantify allostery, but they do not identify the underlying causal molecular mechanisms, which likely involve changes in both conformation and protein dynamics^3,7,73–75^.

However, despite this diversity of possible mechanisms, a conserved principle of allosteric communication appears to be the distance-dependent decay that we have highlighted here. An approximately exponential distance-dependent decay of functional mutational effects is seen in all seven experimental allosteric maps published to date^35–39^ and it is also observed in the PTEN and GCK allosteric maps that we have presented here (Fig. 2). It is also visible when plotting computational predictions of variant fitness after accounting for predicted stability effects (Fig. 5). Distance-dependent decay has been observed for evolutionary conservation in enzyme active sites, for chemical shift perturbations in nuclear magnetic resonance (NMR) experiments, and for energetic couplings in molecular dynamic simulations^65,66,76^. We believe, therefore, that distance-dependent allosteric decay is likely to be a conserved general principle of protein biophysics.

More broadly, the activity–abundance residual framework establishes a foundation for systematically mapping functional communication across the proteome. By quantifying the prevalence and governing principles of distance-dependent decay at scale, this approach will not only reveal the extent of allostery in human proteins but also identify candidate pockets most amenable to therapeutic modulation.

Pervasive and distance-dependent allostery has important implications for human genetics. First, many LOF variants are likely to have allosteric mechanisms of action. Second, our data predict that pathogenic variants are more likely to have allosteric mechanisms when they are located structurally close to active sites. Third, and conversely, drugs targeting non-orthosteric protein pockets are more likely to have strong allosteric effects when they target pockets closer to a protein’s active site. In future work, it will be important to further refine the rules of allostery, allowing improved mechanistic interpretation of variant pathogenicity and prediction of the most effective sites to target to therapeutically modulate protein function.

## Methods

### Data processing for human proteome-wide predictions

Proteome-wide ESM-1v scores were obtained from Zenodo (May 2025)^77^, with detailed methods described in the accompanying publication^78^. AlphaFold predicted structures were downloaded from the AlphaFold Protein Structure Database (June 2025)^79^ and filtered based on criteria used in the reference^24^, excluding proteins shorter than 16 amino acids or longer than 2700 amino acids. This yielded a final dataset of 20,144 unique, unfragmented human proteins, of which 19,288 had matching ESM-1v scores and were retained for downstream analyses.

Stability changes upon mutation (ΔΔG_f_) were predicted for all possible missense mutations using ThermoMPNN v1.0.0^52^ with default settings applied to the AlphaFold-predicted structures.

Protein core positions were identified based on relative solvent-accessible surface area (rSASA), calculated using DSSP with BioPython with default settings^80^. Residues were classified as “core” if they were not predicted to be disordered or part of a binding site and had rSASA <0.2, following criteria from previous publications^81^. Binding and active sites were annotated using proteome-wide predictions from the PIONEER interactome^82^.

Proteins were grouped into functional classes according to the “Function and compartment-based” classification provided by The Human Protein Atlas^83–85^. Clinical variant annotations were obtained from the supplementary materials described above^24^. Variants were originally extracted from ClinVar^54,55,86^ and processed accordingly^26^, retaining only single-nucleotide variants with at least a one-star review status and mapped to GRCh38. For this study, variants labeled pathogenic or likely pathogenic were grouped as “pathogenic”, while those labeled benign or likely benign were grouped as “benign”. Variants with other labels or synonymous mutations were excluded. This yielded 69,909 benign and 32,147 pathogenic clinically annotated variants. ThermoMPNN-predicted missense mutations were classified as: destabilizing if ΔΔG_f_ > 1 kcal/mol, stabilizing if ΔΔG_f_ < –0.5 kcal/mol, following the thresholds defined in the original publication^52^.

A subset of allosteric proteins, listed in Supplemental Table 3, was selected for targeted analyses. These included all full-length human proteins present in two curated datasets: six proteins from the 20-protein benchmark set used to validate the allosteric network model Ohm^87^, and one additional protein, isocitrate dehydrogenase 1 (IDH1), included from the reference^88^. The enzyme prothrombin was excluded because its allosteric regulator functions as an activator rather than an inhibitor. For glucokinase (GCK), annotations of allosteric sites were based on literature reports rather than crystal structure contacts, as its allosteric regulator (MRK) is also an activator. Orthosteric and allosteric residues were defined based on direct atomic contacts with bound substrates or modulators as observed in crystal structures. Residues were considered in contact if their atoms exhibited van der Waals overlap with atoms of the ligand, as determined using the ChimeraX VDW overlap method^89^.

### Data processing for mega-scale, domainome 1.0, VAMP-seq, and SGE datasets

ESM-1v scores for variants in the ‘Mega-scale’ dataset were obtained from the ProteinGym database^90^. For the ‘Domainome 1.0’ dataset, ESM-1v scores were extracted from the supplementary materials of the original publication^28^. ESM-1v scores for ‘VAMP’-seq and SGE variants were derived from the proteome-wide ESM-1v dataset described above. ‘VAMP’-seq abundance fitness data were collected for nine full-length human proteins: PRKN, OCT, CYP2C9, TPMT, ASPA, NUDT15, VKOR, PTEN, and GCK (Supplemental Table 2)^33,61,91–96^. For the GCK dataset, we utilized experimentally measured functional scores and excluded all imputed values.

AlphaMissense predictions were downloaded from the Zenodo repository [https://zenodo.org/records/10813168] in November 2025, and a logit transformation was applied to the raw AlphaMissense scores. ESM-2^51^ scores were generated using the 3-billion parameter model (esm2_t36_3B_UR50D); consistent with the ESM-1v protocol, variant effects were quantified as the log-likelihood ratio between the mutant and wild-type amino acids at a masked position. Vertebrate PhyloP scores were extracted from the CADD^97^ website [https://cadd.gs.washington.edu/download] in January 2025 and aggregated by amino acid substitution to obtain the median conservation scores for each missense variant.

Clinical variant annotations for both the ‘Domainome 1.0’ and ‘VAMP’-seq proteins were obtained from the supplementary data of the reference described above^24^, following the same filtering criteria.

For SGE data, to directly compare the biological signal against predictive noise, we rank-normalized both the experimental SGE scores and ESM-1v predictions to a common 0–1 scale. We then calculated the ‘predictive noise’ as the difference between these normalized values for each variant (Normalized SGE – Normalized ESM) and analyzed whether this deviation followed a spatially coherent, distance-dependent decay characteristic of allostery or the spatial uniformity expected of random model error.

### Variant classification

- ‘Domainome 1.0’ variants were classified based on criteria defined in the original publication. Mutations were considered stabilizing if they had a statistically significant stabilizing effect (*FDR_stabilizing* < 0.1) and a scaled fitness > 0.3. Conversely, mutations were classified as destabilizing if they had a significant destabilizing effect (*FDR_destabilizing* < 0.1) and a scaled fitness <0.
- ‘VAMP’-seq variants were categorized based on abundance thresholds defined by ProteinGym^90^. Mutations falling below the defined abundance cutoff were considered destabilizing.
- ‘Mega-scale’ variants were classified using thresholds established in the original study^27^. Note that the ΔΔG_f_ directionality in this dataset is different relative to others: mutations with ΔΔG_f_ > 1 kcal/mol were classified as stabilizing, while those with ΔΔG_f_ < –1 kcal/mol were classified as destabilizing.

### Data processing for experimental allosteric maps

Analyses for PSD-95-PDZ3 and GRB2-SH3 were performed using variants with corresponding ESM-1v scores, obtained from the human proteome-wide prediction dataset. Orthosteric sites were defined based on published structural annotations^35^. Variants were classified as allosteric if they occurred outside orthosteric sites and exhibited binding ΔΔG (ΔΔG_b_) values greater than or equal to the mean ΔΔG_b_ of orthosteric-site mutations. Evolutionary coupling scores were obtained from the EVcouplings server, and for each position, we calculated the maximum coupling strength (cn) to any residue within the defined orthosteric interface^44,98^.

For PTEN and GCK, analyses were based on variants with both abundance and activity fitness measurements. In PTEN, orthosteric site annotations were manually curated from the literature^99–105^. These included the catalytic loops (arginine loop, residues 35–49; WPD loop, 88–98; P loop, 123–130; TI loop, 159–171), calcium-binding regions (residues 200–212, 226–238, and 258–268), the Cα2 loop (residues 327–335), the membrane-binding helix (residues 151–174), and the PI(4,5)P₂-binding motif (PBM, residues 1–15).

In GCK, orthosteric sites were defined based on annotated ligand-contacting residues described in previous structural studies^106–109^. These included glucose-binding residues (positions 151–153, 168–169, 204–206, 225–231, 254–258, 287, and 290) and ATP-binding residues (positions 78–85, 295–296, 331–333, 336, and 410–416).

For BRCA1, we defined orthosteric sites within the RING domain as residues contacting BARD1-RING (3, 6–8, 10–11, 14–15, 17–18, 21–22, 38, 76–79, 81–82, 85, 88–89, 92–93, 96–97) or coordinating Zn^2+^ ions (24, 27, 39, 41, 44, 47, 61, 64). In the BRCA1-BRCT domain, sites were defined as residues comprising the BACH1 binding interface (1655–1658, 1690–1691, 1696, 1698–1702, 1740–1741, 1774–1775, 1811, 1813, 1836, 1839).

For RAD51C, orthosteric sites were defined as the ATP-binding Walker A and Walker B motifs (residues 125–132 and 238–242) and the nuclear localization sequence^110^ (residues 366–370). For BAP1, orthosteric sites were defined as the catalytic residues^111^ (85, 91, 169, 184).

For PTEN, GCK, and the SGE genes, updated ClinVar data were downloaded from the NCBI ClinVar website (https://www.ncbi.nlm.nih.gov/clinvar/) in January 2026 and processed using the same filtering criteria described above.

### Statistical analysis and data visualization

Linear regression models were used to analyze proteome-wide variant effect predictions, large-scale abundance datasets, and individual protein domains with experimentally mapped allosteric mutations. To evaluate the relative contributions of folding ΔΔG_f_ and binding ΔΔG_b_ to ESM-1v scores, both ordinary linear regression and linear mixed-effects models were employed. Linear regression was performed using the lm() function from the stats package in R, while mixed-effects models were fitted using the lmer() function from the lme4 package^112,113^. As results from both approaches were highly consistent (Supplemental Tables 4–7), linear regression outcomes are reported for interpretability. Adjusted R² values were extracted directly from the summary output of the fitted linear models in R. To estimate variability and provide robust confidence intervals, adjusted R² was also bootstrapped using 1000 resampling iterations. Model diagnostics included residual and prediction analysis, and model comparisons were performed using ANOVA, with F-statistics and p-values reported.

LOESS residual analyses were performed using only mutations located outside orthosteric sites. LOESS fitting was conducted with the loess() function in R, using a span of 0.7 and the family = “symmetric” setting. For all proteins, distances were calculated as the minimum distance between any side-chain heavy atom of the residue and the ligand or orthosteric site.

To generate abundance-matched controls, we performed bootstrap resampling using all non-pathogenic variants as the sampling pool. Variants were grouped into fixed-width abundance bins (bin size = 0.5) spanning the full range of experimentally measured abundance scores. For each bootstrap iteration (*n* = 1000), we generated a matched null dataset by replacing each pathogenic variant with a randomly sampled non-pathogenic variant from the same abundance bin. Statistical significance between pathogenic variants and the abundance-matched null model was assessed using an empirical p-value, defined as the fraction of bootstrap iterations where the median residual of the null distribution was lower than or equal to the observed median residual of the pathogenic variants. Additionally, differences between benign and pathogenic variants were assessed using a Wilcoxon rank-sum test.

To quantify the distance-dependent decay of mutational effects, nonlinear least-squares regression was applied to the LOESS residuals. The allosteric decay model included two parameters: the decay rate (−b) and the x-intercept (a). This fitting was performed using only mutations with negative residuals that fell below the LOESS curve. Nonlinear regression was conducted with the nls() function in R, using initial parameter values of a = 1 and b = 0.1. 95% confidence intervals were calculated by bootstrapping the model parameter with 1000 replicates.

All statistical analyses were performed in R version 4.5.0^113^. Experimental schematics were created using the NIH BioArt Source, while all other plots were generated using the ggplot2 package version 4.0.3^114^. Three-dimensional protein structure visualizations were rendered using ChimeraX version 1.6.1^89^.

### Reporting summary

Further information on research design is available in the Nature Portfolio Reporting Summary linked to this article.

## Supplementary information

Supplementary Information

Reporting Summary

Transparent Peer Review file

## Source data

Source Data

## Data Availability

Proteome-wide predictions of protein stability changes and corresponding ESM-1v scores generated in this study have been deposited in the Zenodo database under accession code 18386427 as Supplementary Table 1. Raw ThermoMPNN predictions of protein stability changes generated in this study are available at Zenodo under accession code 18381534. Large-scale experimental measurements of protein abundance changes across human domains, including datasets from the ‘Mega-scale’ and ‘Domainome’ studies, are accessible via their respective supplementary materials. ‘VAMP’-seq abundance data are available from MaveDB [10.1186/s13059-019-1845-6] and ProteinGym [10.1101/2023.12.07.570727]^90,115^. Experimental ∆∆G values for PSD95-PDZ3 and GRB2-SH3 domains, and experimental measurements of abundance and activity-based fitness for PTEN and GCK, and SGE scores, are available through the original studies cited in the references. PDB codes include 1D5R, 1V4S, 1T29, 1JM7, 1BE9, 2VWF, 4RQK, 1PTY, 2HBQ, 2E9N, 6IO0. Source data are provided with this paper.

## Code availability

All code and metadata to reproduce the analyses are hosted on GitHub [https://github.com/lehner-lab/allostery_pathogenicity].
