# Supplementary material for: Allostery is a widespread cause of loss-of-function variant pathogenicity
Source: Nat Commun. 2026 Jun 22;17:7841. doi: 10.1038/s41467-026-74517-8 (PMC13439481; doi:10.1038/s41467-026-74517-8)
Supplement: Supplementary file 1 [file 41467_2026_74517_MOESM1_ESM.pdf]

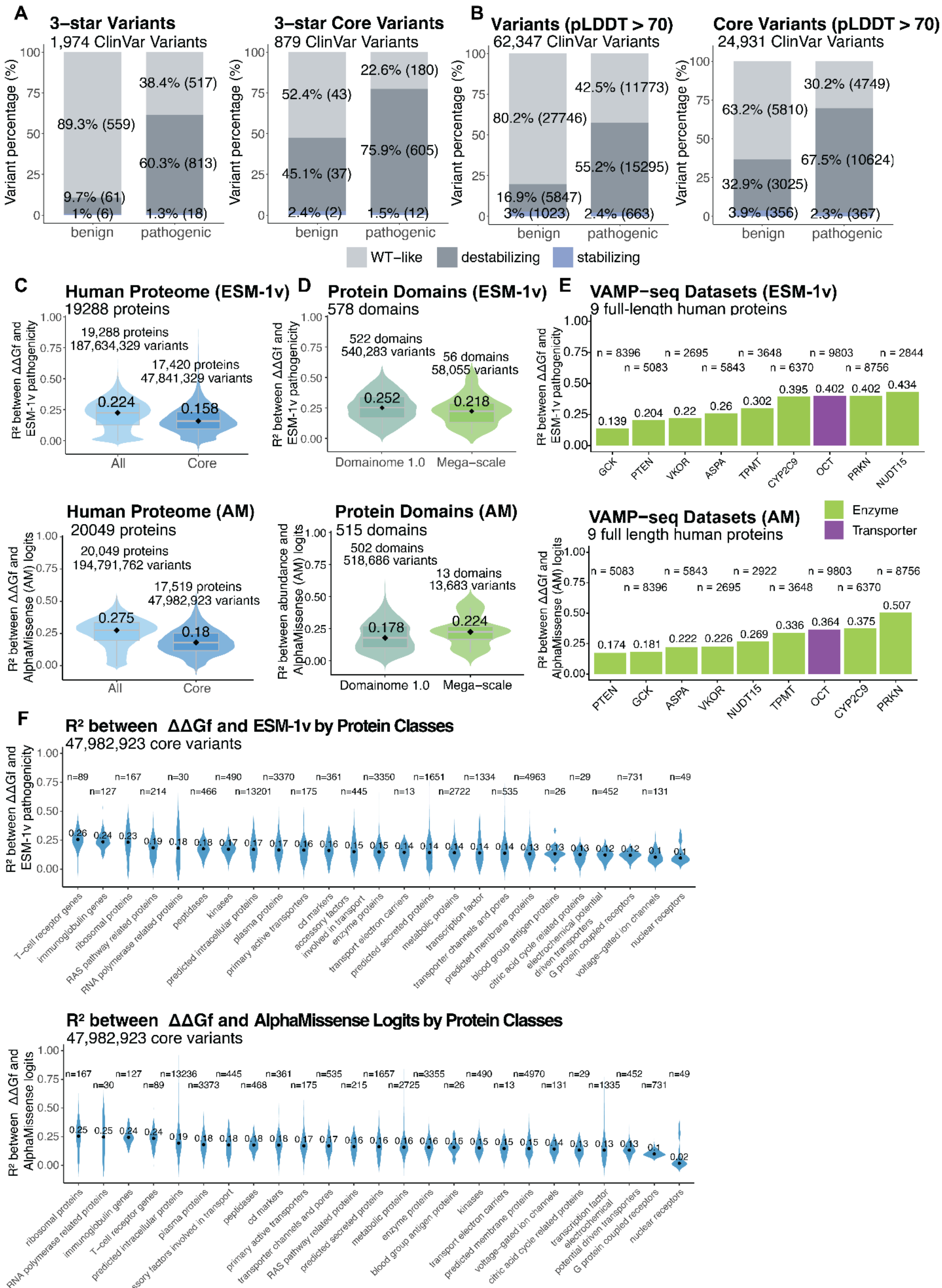
A
3−star Variants
1,974 ClinVar Variants
3−star Core Variants
879 ClinVar Variants
B
Variants (pLDDT > 70)
62,347 ClinVar Variants
Core Variants (pLDDT > 70)
24,931 ClinVar Variants
Variant percentage (%)
89.3% (559)
9.7% (61)
1% (6)
38.4% (517)
60.3% (813)
1.3% (18)
52.4% (43)
45.1% (37)
2.4% (2)
22.6% (180)
75.9% (605)
1.5% (12)
80.2% (27746)
16.9% (5847)
3% (1023)
42.5% (11773)
55.2% (15295)
2.4% (663)
63.2% (5810)
32.9% (3025)
3.9% (356)
30.2% (4749)
67.5% (10624)
2.3% (367)
benign
pathogenic
WT−like
destabilizing
stabilizing
C
Human Proteome (ESM-1v)
19288 proteins
19,288 proteins
187,634,329 variants
17,420 proteins
47,841,329 variants
0.224
0.158
All
Core
R² between ΔΔGf and ESM-1v pathogenicity
D
Protein Domains (ESM-1v)
578 domains
522 domains
540,283 variants
56 domains
58,055 variants
0.252
0.218
Domainome 1.0
Mega-scale
E
VAMP−seq Datasets (ESM-1v)
9 full-length human proteins
n = 8396
n = 5083
n = 2695
n = 5843
n = 3648
n = 6370
n = 9803
n = 8756
n = 2844
0.139
0.204
0.22
0.26
0.302
0.395
0.402
0.402
0.434
GCK
PTEN
VKOR
ASPA
TPMT
CYP2C9
OCT
PRKN
NUDT15
Human Proteome (AM)
20049 proteins
20,049 proteins
194,791,762 variants
17,519 proteins
47,982,923 variants
0.275
0.18
All
Core
R² between ΔΔGf and AlphaMissense (AM) logits
Protein Domains (AM)
515 domains
502 domains
518,686 variants
13 domains
13,683 variants
0.178
0.224
Domainome 1.0
Mega-scale
R² between abundance and AlphaMissense (AM) logits
VAMP−seq Datasets (AM)
9 full length human proteins
Enzyme
Transporter
n = 5083
n = 8396
n = 5843
n = 2695
n = 2922
n = 3648
n = 9803
n = 6370
n = 8756
0.174
0.181
0.222
0.226
0.269
0.336
0.364
0.375
0.507
PTEN
GCK
ASPA
VKOR
NUDT15
TPMT
OCT
CYP2C9
PRKN
F
R² between ΔΔGf and ESM-1v by Protein Classes
47,982,923 core variants
R² between ΔΔGf and ESM-1v pathogenicity
n=89
n=127
n=167
n=214
n=30
n=466
n=490
n=13201
n=3370
n=175
n=361
n=445
n=3350
n=13
n=1651
n=2722
n=1334
n=535
n=4963
n=26
n=29
n=452
n=731
n=131
n=49
T-cell receptor genes
immunoglobulin genes
ribosomal proteins
RAS pathway related proteins
RNA polymerase related proteins
peptidases
kinases
predicted intracellular proteins
plasma proteins
primary active transporters
cd markers
accessory factors involved in transport
enzyme proteins
transport electron carriers
predicted secreted proteins
metabolic proteins
transcription factor
transporter channels and pores
predicted membrane proteins
blood group antigen proteins
citric acid cycle related proteins
electrochemical potential driven transporters
G protein coupled receptors
voltage-gated ion channels
nuclear receptors
R² between ΔΔGf and AlphaMissense Logits by Protein Classes
47,982,923 core variants
R² between ΔΔGf and AlphaMissense logits
n=167
n=30
n=127
n=89
n=13236
n=3373
n=445
n=468
n=361
n=175
n=535
n=215
n=1657
n=2725
n=3355
n=26
n=490
n=13
n=4970
n=131
n=29
n=1335
n=452
n=731
n=49
ribosomal proteins
RNA polymerase related proteins
immunoglobulin genes
T-cell receptor genes
predicted intracellular proteins
plasma proteins
accessory factors involved in transport
peptidases
cd markers
primary active transporters
transporter channels and pores
RAS pathway related proteins
predicted secreted proteins
metabolic proteins
enzyme proteins
blood group antigen proteins
kinases
transport electron carriers
predicted membrane proteins
voltage-gated ion channels
citric acid cycle related proteins
transcription factor
electrochemical potential driven transporters
G protein coupled receptors
nuclear receptors

Fig. S1: Stability explains only a modest fraction of pathogenicity across diverse datasets and predictors. A. Classification of 3-star ClinVar missense variants into three categories: WT-like (light grey), destabilizing (dark grey), and stabilizing (blue), based on predicted $\Delta\Delta G_f$. Left: All variants. Right: Core variants. **B.** Classification of ClinVar variants mapped to high-confidence positions in the AlphaFold predicted structures (pLDDT > 70). Left: All variants. Right: Core variants **C**. Distribution of R^2 values from linear regression between predicted stability and predicted pathogenicity scores across the human proteome. Comparisons are shown for ESM-1v (top) and AlphaMissense (AM) (bottom), calculated for all variants and restricted to protein core variants. Median R^2 values are labeled. **D.** R^2 between measured abundance and pathogenicity scores evaluated on large-scale experimental abundance datasets: 'Domainome 1.0' (cellular abundance) and 'Mega-scale' (protease sensitivity). Top: ESM-1v. Bottom: AlphaMissense (AM). **E**. R^2 values between measured abundance and pathogenicity scores for nine full-length proteins profiled using 'VAMP'-seq (Supplemental Table 2). Bars are colored by protein class. **F.** Distribution of R^2 values between predicted stability and pathogenicity scores, stratified by protein class (defined by the Human Protein Atlas).

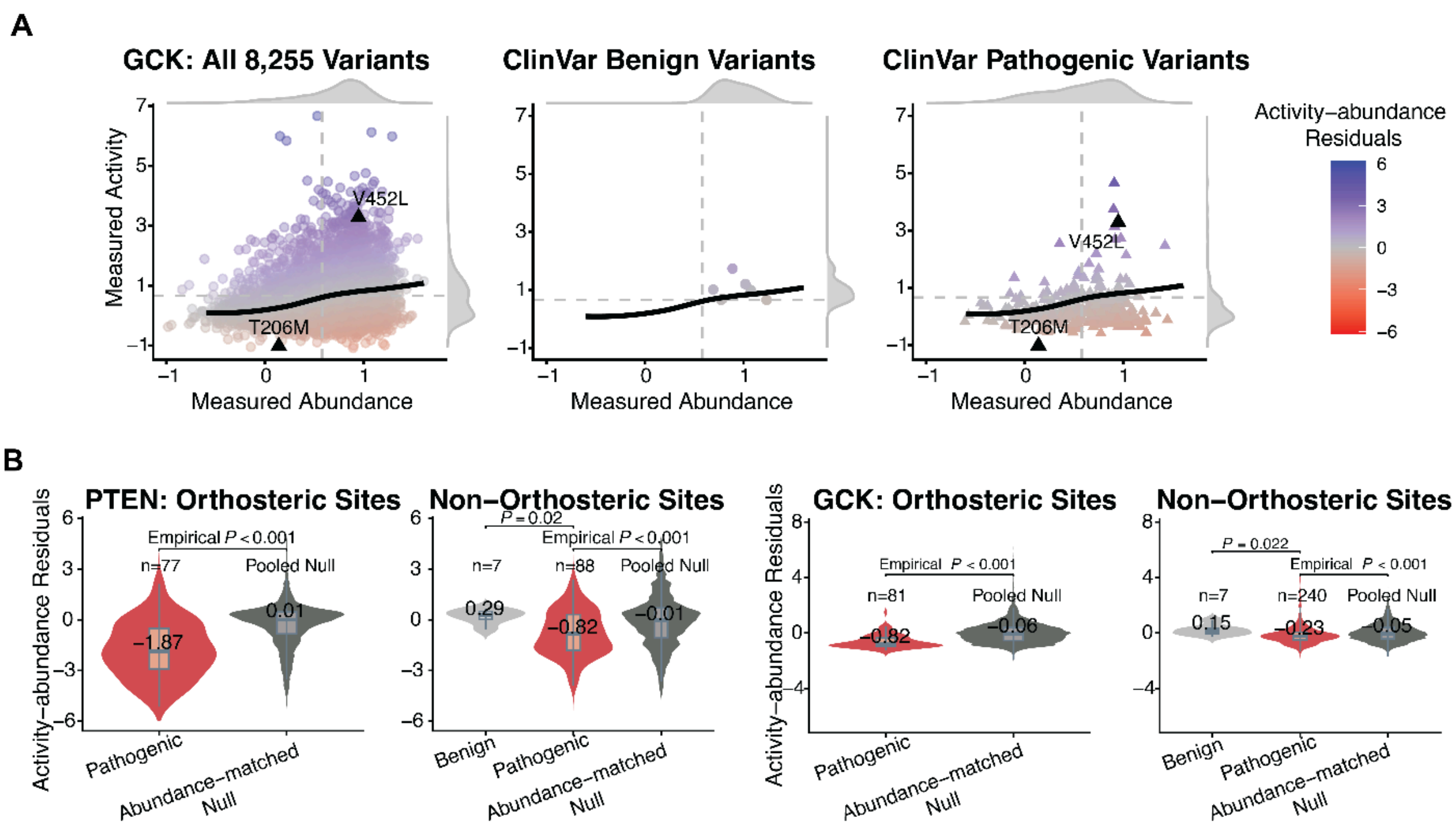

Fig. S2: Activity–abundance residual analysis for GCK and stratification by structural location. A. Scatter plots of measured protein abundance (Abundance PCA) versus glucokinase activity (yeast complementation) for 8,255 GCK variants. A LOESS regression curve (black line) models the overall trend. Left: All variants. Center: ClinVar Benign variants. Right: ClinVar Pathogenic variants. Grey dashed lines indicate wild-type abundance (0.58) and activity (0.66) values. The canonical gain-of-function variant (V452L) and loss-of-function variant (T206M) are highlighted. **B.** Distributions of activity–abundance residuals for ClinVar benign, pathogenic, and abundance-matched control variants in PTEN (left) and GCK (right). Activity-abundance residuals are stratified by structural location (orthosteric vs. non-orthosteric sites). For all distributions, the violins show the kernel density of all values. Within each violin, an embedded boxplot indicates the median (center horizontal line; values labelled) and the interquartile range (IQR), with whiskers extending to 1.5× the IQR; an overlaid vertical line spans the 2.5th–97.5th percentiles (central 95% of values). To generate the abundance-matched null distribution, variants were first binned by their measured protein abundance (0.5-unit window). For each pathogenic variant, a residual score was randomly sampled from the pool of non-pathogenic variants residing in the same abundance bin (1,000 bootstrap iterations). Deviation of pathogenic variants from this null expectation was assessed via a one-sided empirical P-value. Comparisons between Benign and Pathogenic variants were performed using a one-sided Wilcoxon rank-sum test.

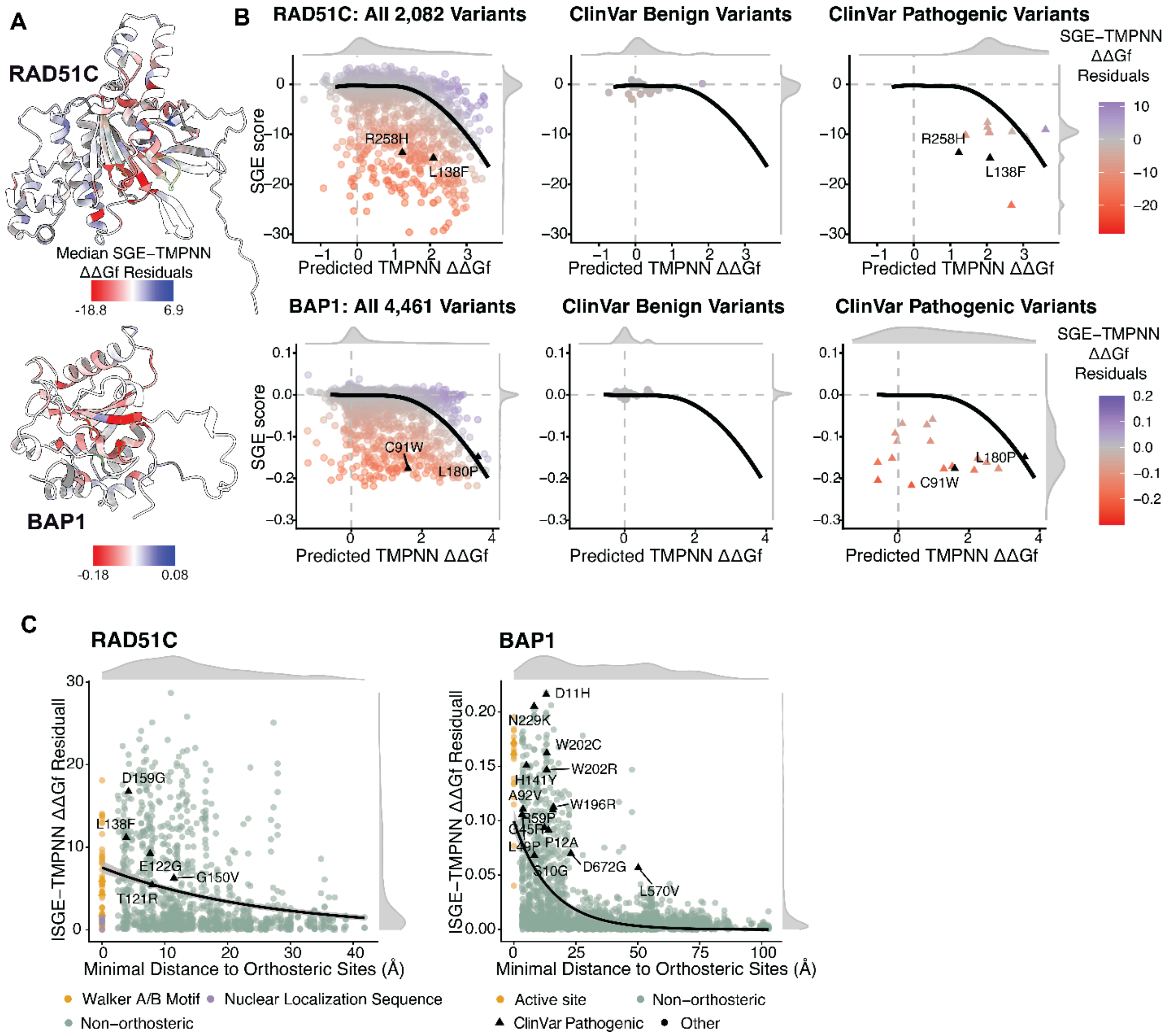

Fig. S3: Functional residual analysis and distance-dependent decay in RAD51C and BAP1. A. AlphaFold2 predicted structures of RAD51C (top) and the BAP1 UCH domain (bottom), colored by the median SGE–ThermoMPNN $\Delta\Delta G_f$ residuals. Orthosteric sites are highlighted with neon green outlines (RAD51C: Walker A and B motifs; BAP1: catalytic active sites). **B.** Scatter plots of experimental function (SGE score) versus predicted ThermoMPNN $\Delta\Delta G_f$. A LOESS regression (black line) models the expected relationship between stability and function. Left: All variants. Center: ClinVar Benign variants. Right: ClinVar Pathogenic variants. Top: RAD51C. Bottom: BAP1. Example pathogenic variants are highlighted. **C.** Distance-dependent decay of functional effects for RAD51C (left) and BAP1 (right). The absolute magnitude of the SGE–ThermoMPNN $\Delta\Delta G_f$ was plotted against the minimal side-chain heavy atom distance to the orthosteric sites, with 95% bootstrap confidence intervals shown as grey shading. ClinVar pathogenic variants (≥1 star) located outside of orthosteric sites are marked with black triangles.

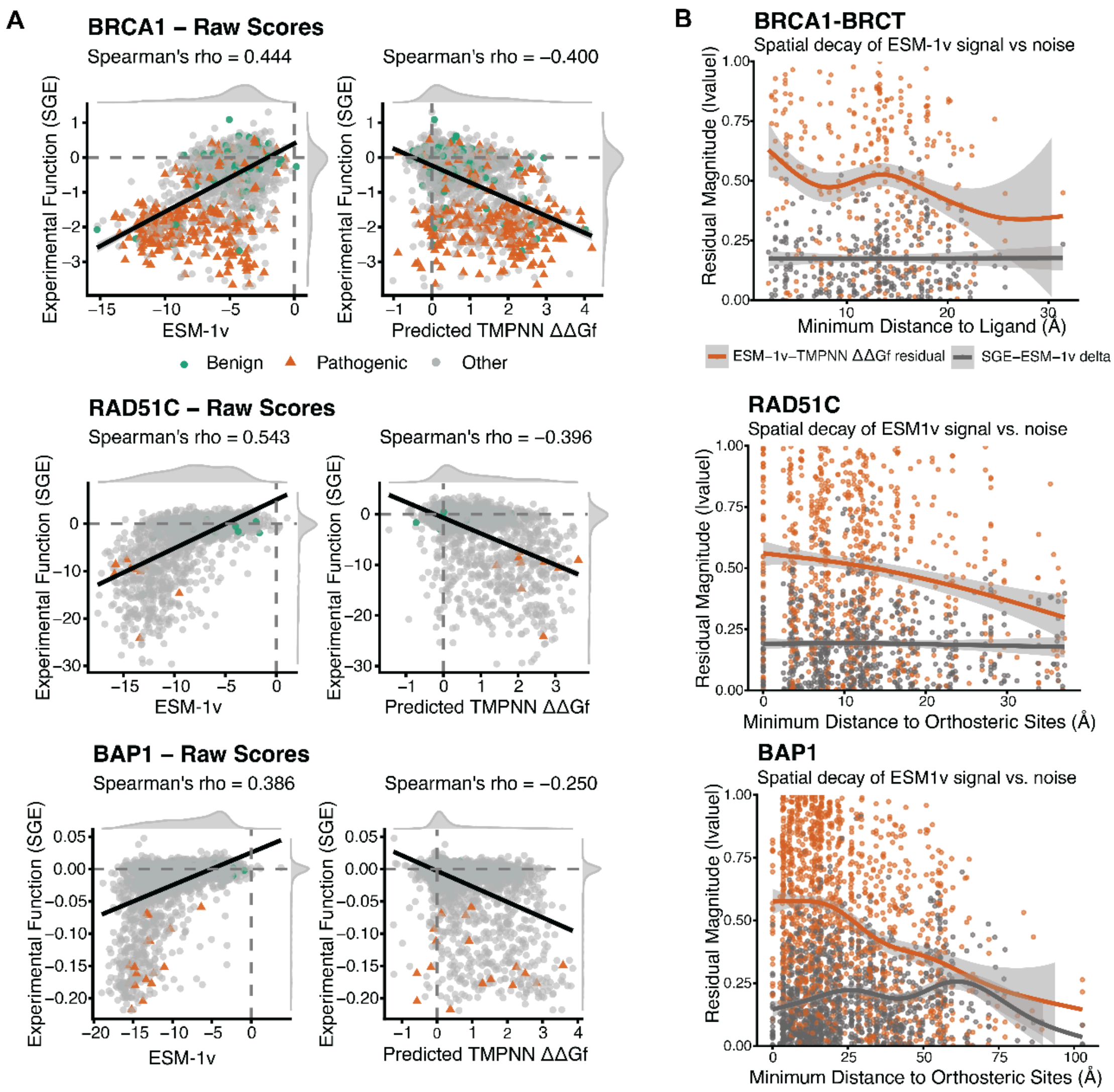

Fig S4: Validation of computational residuals against SGE ground truth and spatial signal-to-noise analysis. A. Scatter plots comparing experimental function (SGE) against ESM-1v (left) and predicted stability (right) for BRCA1 (top), RAD51C (middle), and BAP1 (bottom). ClinVar pathogenic variants are denoted as orange triangles and benign variants as green circles. **B.** Spatial analysis of signal versus noise. The absolute magnitude of the predicted biological signal (orange: rank-normalized ESM-1v–ThermoMPNN $\Delta\Delta G_f$ residual) and the model error (grey: rank-normalized SGE–ESM-1v delta) was plotted against the minimum heavy atom distance from the functional site/ligand for BRCA1-BRCT (top), RAD51C (middle), and BAP1 (bottom). Trend lines represent Generalized Additive Models (GAM) fitted to capture non-linear spatial decay; shaded regions indicate the 95% confidence interval of the fit.

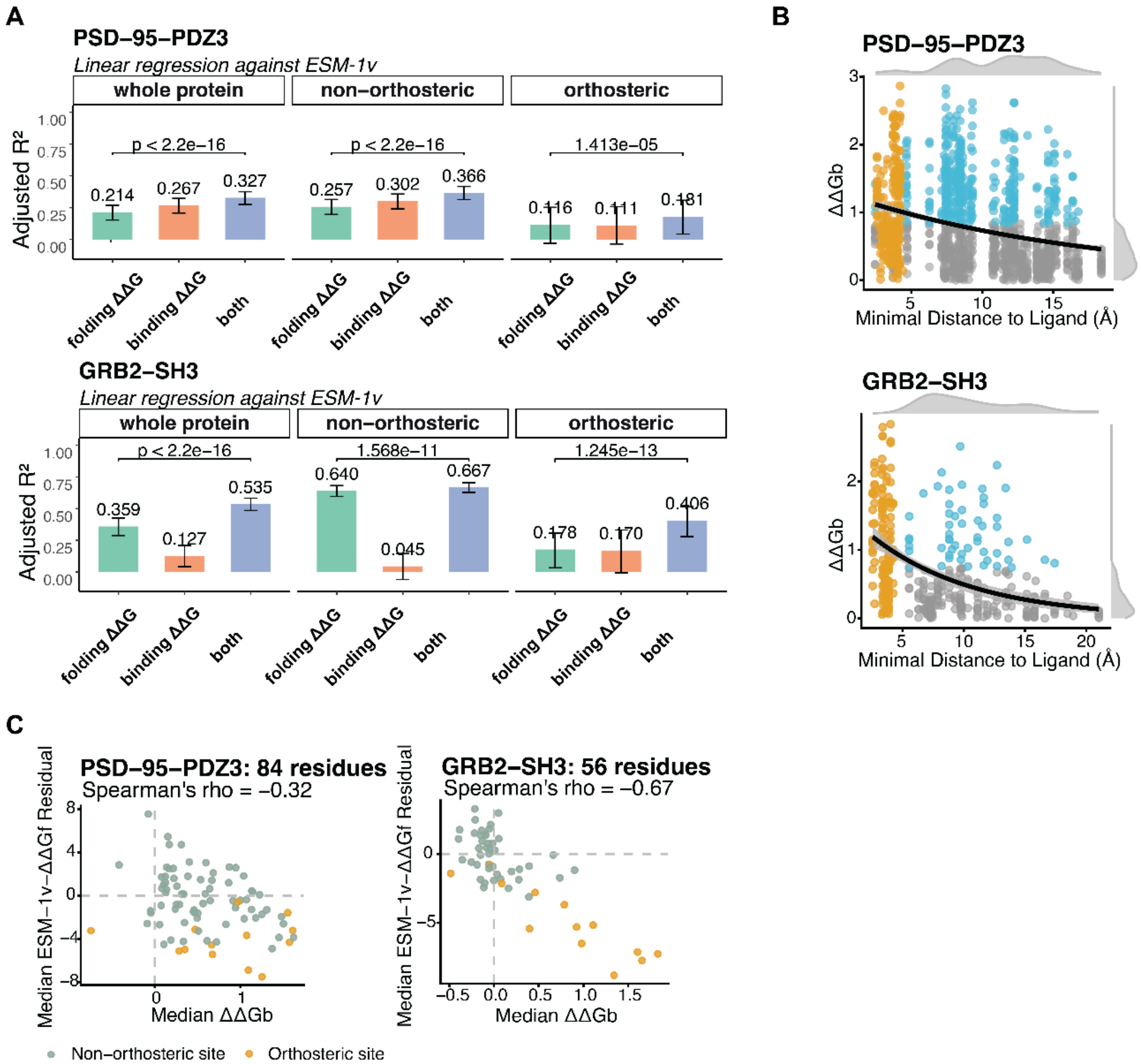

Fig. S5: Contributions of folding and binding free energies to predicted ESM-1v evolutionary fitness in PSD-95-PDZ3 and GRB2-SH3. A. Linear regression models quantifying the variance in ESM-1v scores explained by measured $\Delta\Delta G_f$ (folding free energy changes), $\Delta\Delta G_b$ (binding free energy changes), or both, across orthosteric and non-orthosteric sites. Adjusted R^2 values are shown with bootstrapped confidence intervals. Statistical significance was evaluated using ANOVA to compare each baseline model to the corresponding full model including both energy terms. Top: Results for PSD-95-PDZ3. Bottom: Results for GRB2-SH3. **B.** Distance-dependent decay of experimentally measured binding free energies. The absolute magnitude of the residual was plotted against the minimal distance to the ligand. An exponential decay model (black line) was fitted to the data, with 95% bootstrap confidence intervals shown as grey shading. Variants are colored by site type (orange: orthosteric; blue: allosteric; grey: other). Top: PSD-95-PDZ3. Bottom: GRB2-SH3. **C.** Scatter plots showing the median ESM-1v–measured $\Delta\Delta G_f$ residual plotted against the experimentally measured binding free energy change for PSD-95 PDZ3 (left) and GRB2-SH3 (right).

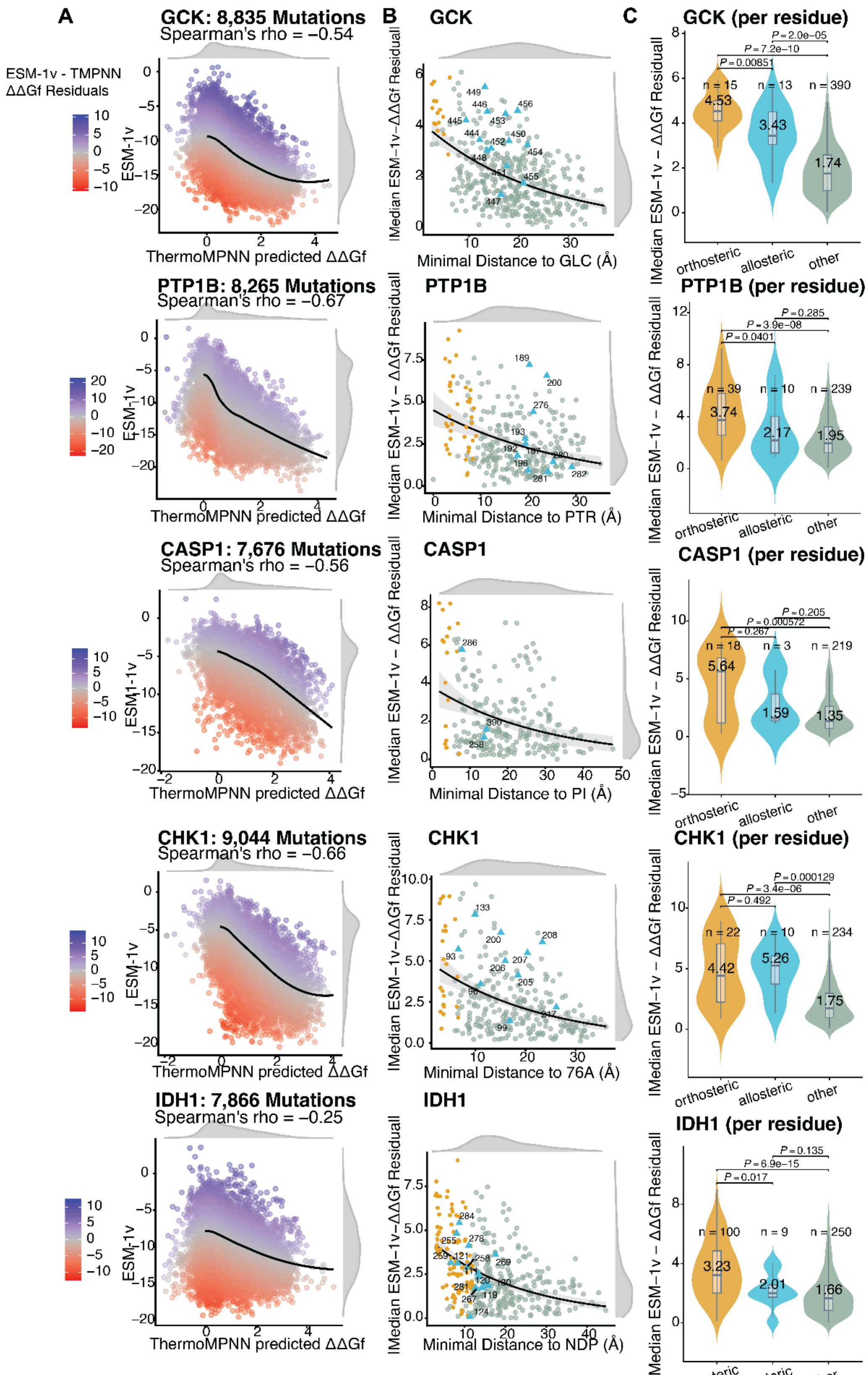
A
B
C
ESM-1v - TMPNN ΔΔGf Residuals
GCK: 8,835 Mutations
Spearman's rho = −0.54
ESM-1v
ThermoMPNN predicted ΔΔGf
GCK
|Median ESM−1v−ΔΔGf Residual|
Minimal Distance to GLC (Å)
GCK (per residue)
|Median ESM−1v − ΔΔGf Residual|
P = 2.0e−05
P = 7.2e−10
P = 0.00851
n = 15
n = 13
n = 390
4.53
3.43
1.74
orthosteric
allosteric
other
PTP1B: 8,265 Mutations
Spearman's rho = −0.67
PTP1B
Minimal Distance to PTR (Å)
PTP1B (per residue)
P = 0.285
P = 3.9e−08
P = 0.0401
n = 39
n = 10
n = 239
3.74
2.17
1.95
CASP1: 7,676 Mutations
Spearman's rho = −0.56
CASP1
Minimal Distance to PI (Å)
CASP1 (per residue)
P = 0.205
P = 0.000572
P = 0.267
n = 18
n = 3
n = 219
5.64
1.59
1.35
CHK1: 9,044 Mutations
Spearman's rho = −0.66
CHK1
Minimal Distance to 76A (Å)
CHK1 (per residue)
P = 0.000129
P = 3.4e−06
P = 0.492
n = 22
n = 10
n = 234
4.42
5.26
1.75
IDH1: 7,866 Mutations
Spearman's rho = −0.25
IDH1
Minimal Distance to NDP (Å)
IDH1 (per residue)
P = 0.135
P = 6.9e−15
P = 0.017
n = 100
n = 9
n = 250
3.23
2.01
1.66

Fig. S6: Distance-dependent decay of predicted functional effects in diverse allosteric enzymes. A. Scatter plots of ThermoMPNN-predicted $\Delta\Delta G_f$ versus ESM-1v scores for GCK, PTP1B, CASP1, CHK1, and IDH1. A LOESS regression (black line) models the trend for each protein. **B.** Per-residue relationship between the absolute median ESM-1v–ThermoMPNN $\Delta\Delta G_f$ residual and the minimal distance to the active site ligand (indicated in x-axis labels). An exponential decay model (black curve) was fitted to the data, with 95% bootstrap confidence intervals shown as grey shading. Known allosteric regulator contact sites are labeled as cyan triangles. **C.** Distributions of per-site absolute median residuals for orthosteric, allosteric, and other sites. Significance values were calculated using the Wilcoxon rank-sum test. For all distributions, the violins show the kernel density of all values. Within each violin, an embedded boxplot indicates the median (center horizontal line; values labelled) and the interquartile range (IQR), with whiskers extending to 1.5× the IQR; an overlaid vertical line spans the 2.5th–97.5th percentiles (central 95% of values). Comparisons were performed using a one-sided Wilcoxon rank-sum test.

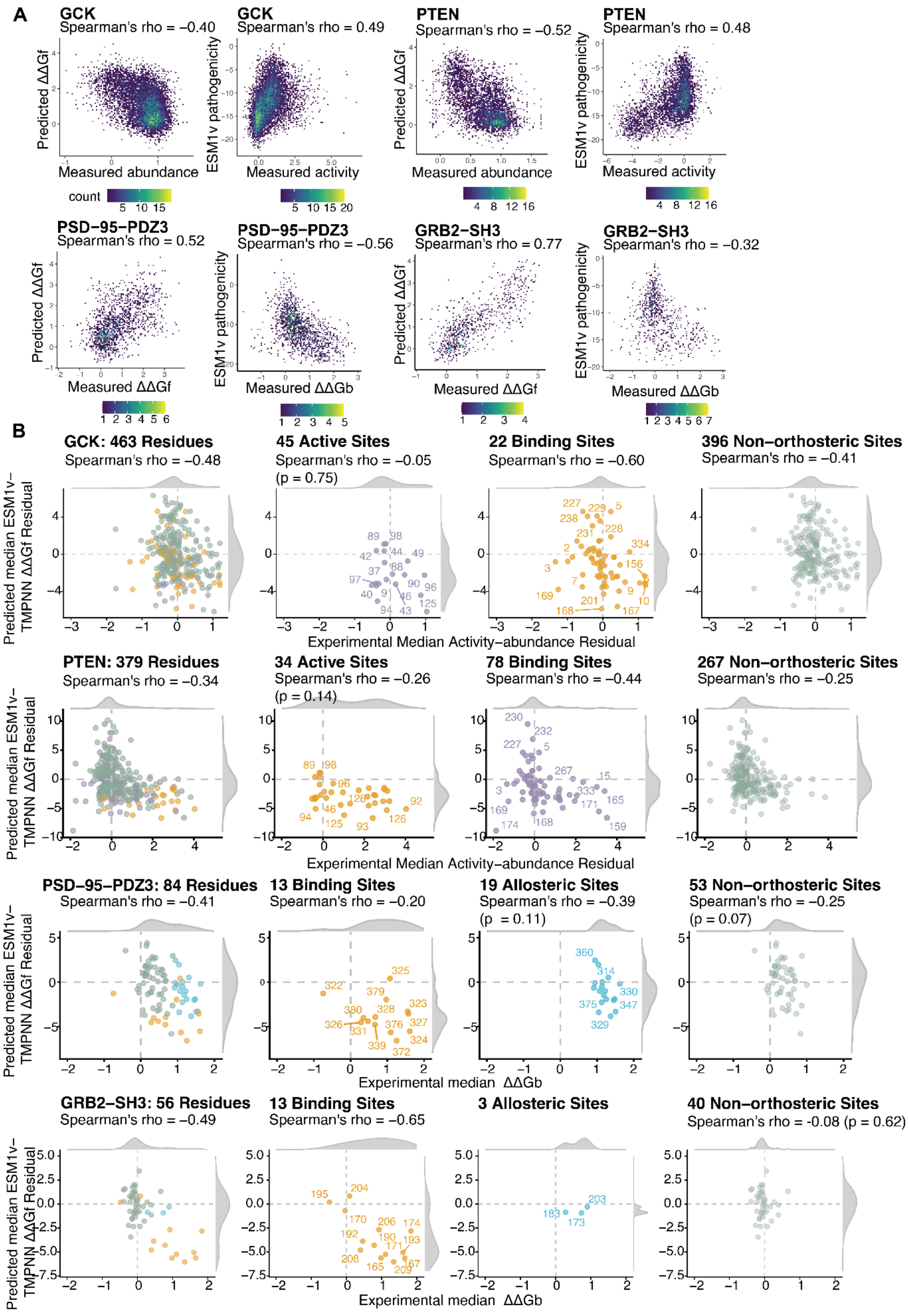
A
GCK
Spearman's rho = −0.40
GCK
Spearman's rho = 0.49
PTEN
Spearman's rho = −0.52
PTEN
Spearman's rho = 0.48
Predicted ΔΔGf
ESM1v pathogenicity
Measured abundance
Measured activity
count
PSD−95−PDZ3
Spearman's rho = 0.52
PSD−95−PDZ3
Spearman's rho = −0.56
GRB2−SH3
Spearman's rho = 0.77
GRB2−SH3
Spearman's rho = −0.32
Measured ΔΔGf
Measured ΔΔGb
B
GCK: 463 Residues
Spearman's rho = −0.48
45 Active Sites
Spearman's rho = −0.05
(p = 0.75)
22 Binding Sites
Spearman's rho = −0.60
396 Non−orthosteric Sites
Spearman's rho = −0.41
Predicted median ESM1v−TMPNN ΔΔGf Residual
Experimental Median Activity−abundance Residual
PTEN: 379 Residues
Spearman's rho = −0.34
34 Active Sites
Spearman's rho = −0.26
(p = 0.14)
78 Binding Sites
Spearman's rho = −0.44
267 Non−orthosteric Sites
Spearman's rho = −0.25
Experimental Median Activity−abundance Residual
PSD−95−PDZ3: 84 Residues
Spearman's rho = −0.41
13 Binding Sites
Spearman's rho = −0.20
19 Allosteric Sites
Spearman's rho = −0.39
(p = 0.11)
53 Non−orthosteric Sites
Spearman's rho = −0.25
(p = 0.07)
Experimental median ΔΔGb
GRB2−SH3: 56 Residues
Spearman's rho = −0.49
13 Binding Sites
Spearman's rho = −0.65
3 Allosteric Sites
40 Non−orthosteric Sites
Spearman's rho = -0.08 (p = 0.62)
Experimental median ΔΔGb

Fig. S7: Benchmarking computational residuals against experimental measurements across proteins. **A**. Density scatter plots showing the relationship between (left) ThermoMPNN-predicted $\Delta\Delta G_f$ and experimentally measured abundance, and (right) ESM-1v scores and experimentally measured activity for missense variants in GCK, PTEN, PSD-95-PDZ3, and GRB2-SH3. Point density is color-coded, and Spearman correlation coefficients (ρ) are shown for each plot. **B**. Per-residue comparison between experimentally derived activity–abundance residuals and computationally derived ESM-1v–$\Delta\Delta G_f$ residuals in GCK, PTEN, PSD95-PDZ3, and GRB2-SH3. Each point represents a single residue, colored by site class. For panels with insignificant Spearman correlations, P values are shown.

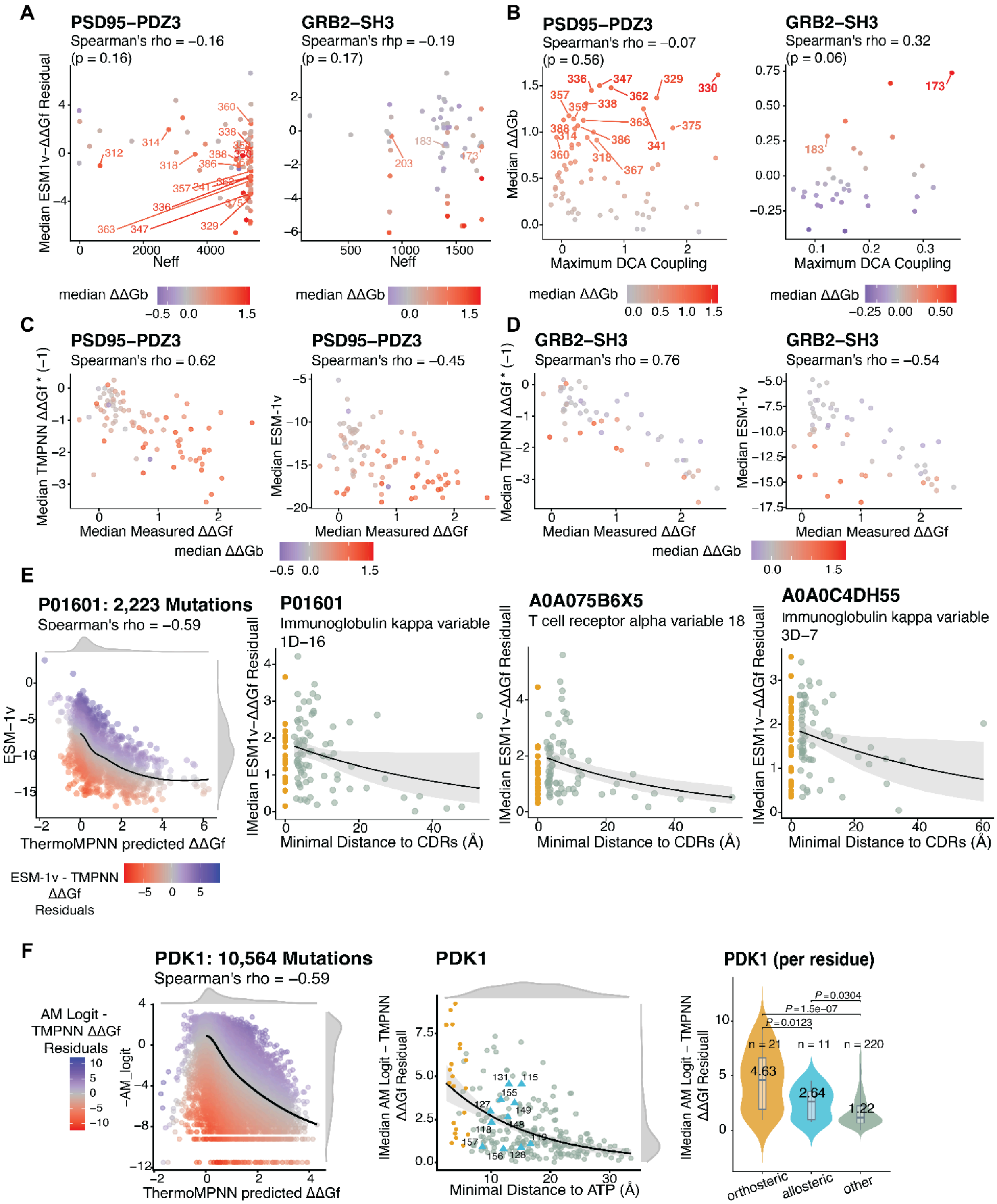

A
PSD95–PDZ3
Spearman's rho = −0.16
(p = 0.16)
Median ESM1v−ΔΔGf Residual
Neff
GRB2–SH3
Spearman's rhp = −0.19
(p = 0.17)
Neff
median ΔΔGb
B
PSD95–PDZ3
Spearman's rho = −0.07
(p = 0.56)
Median ΔΔGb
Maximum DCA Coupling
GRB2–SH3
Spearman's rho = 0.32
(p = 0.06)
Maximum DCA Coupling
median ΔΔGb
C
PSD95–PDZ3
Spearman's rho = 0.62
Median TMPNN ΔΔGf * (−1)
Median Measured ΔΔGf
PSD95–PDZ3
Spearman's rho = −0.45
Median ESM-1v
Median Measured ΔΔGf
median ΔΔGb
D
GRB2–SH3
Spearman's rho = 0.76
Median TMPNN ΔΔGf * (−1)
Median Measured ΔΔGf
GRB2–SH3
Spearman's rho = −0.54
Median ESM-1v
Median Measured ΔΔGf
median ΔΔGb
E
P01601: 2,223 Mutations
Spearman's rho = −0.59
ESM−1v
ThermoMPNN predicted ΔΔGf
ESM-1v - TMPNN ΔΔGf Residuals
P01601
Immunoglobulin kappa variable 1D−16
Median ESM1v−ΔΔGf Residual
Minimal Distance to CDRs (Å)
A0A075B6X5
T cell receptor alpha variable 18
Median ESM1v−ΔΔGf Residual
Minimal Distance to CDRs (Å)
A0A0C4DH55
Immunoglobulin kappa variable 3D−7
Median ESM1v−ΔΔGf Residual
Minimal Distance to CDRs (Å)
F
PDK1: 10,564 Mutations
Spearman's rho = −0.59
AM Logit - TMPNN ΔΔGf Residuals
−AM_logit
ThermoMPNN predicted ΔΔGf
PDK1
Median AM Logit − TMPNN ΔΔGf Residual
Minimal Distance to ATP (Å)
PDK1 (per residue)
Median AM Logit − TMPNN ΔΔGf Residual
P = 0.0304
P = 1.5e−07
P = 0.0123
n = 21
n = 11
n = 220
4.63
2.64
1.22
orthosteric
allosteric
other

Fig. S8: Coevolutionary signals, predictor validation, and immunoglobulin domain analysis. A. Scatter plots showing the relationship between positional effective number of sequences (N_{eff}) and median ESM-1v–ThermoMPNN $\Delta\Delta G_f$ residuals in PSD95-PDZ3 (left) and GRB2-SH3 (right). Points are colored by median experimental binding free energy ($\Delta\Delta G_b$), and Spearman correlation coefficients and P values are shown. **B.** Scatter plots showing the relationship between maximum DCA coupling scores (to orthosteric sites) and median experimental binding free energy in PSD95-PDZ3 (left) and GRB2-SH3 (right). **C.** Scatter plots showing the relationship between (left) ThermoMPNN $\Delta\Delta G_f$ (inverted) and experimentally measured $\Delta\Delta G_f$, and (right) ESM-1v scores and experimentally measured ΔΔGf for PSD95-PDZ3. **D.** Scatter plots showing the relationship between (left) ThermoMPNN $\Delta\Delta G_f$ (inverted) and experimentally measured $\Delta\Delta G_f$, and (right) ESM-1v scores and experimentally measured $\Delta\Delta G_f$ for GRB2-SH3. **E.** (Left) Density scatter plot showing the relationship between ESM-1v scores and ThermoMPNN $\Delta\Delta G_f$ for Ig kappa chain P01601. (Right) Absolute median ESM-1v–$\Delta\Delta G_f$ residuals plotted against minimal distance to Complementarity Determining Regions (CDRs) for three proteins (P01601, A0A075B6X5, A0A0C4DH55), with 95% bootstrap confidence intervals shown as grey shading. **F.** Validation using AlphaMissense on PDK1. (Left) Density scatter plot showing the relationship between AlphaMissense logit scores and ThermoMPNN $\Delta\Delta Gf_f$ (Middle) Absolute median AlphaMissense–ThermoMPNN $\Delta\Delta G_f$ residuals plotted against distance to the ATP binding site, with 95% bootstrap confidence intervals shown as grey shading. (Right) Violin plots showing the distribution of residuals for orthosteric, allosteric, and other sites. Comparisons were performed using a one-sided Wilcoxon rank-sum test.

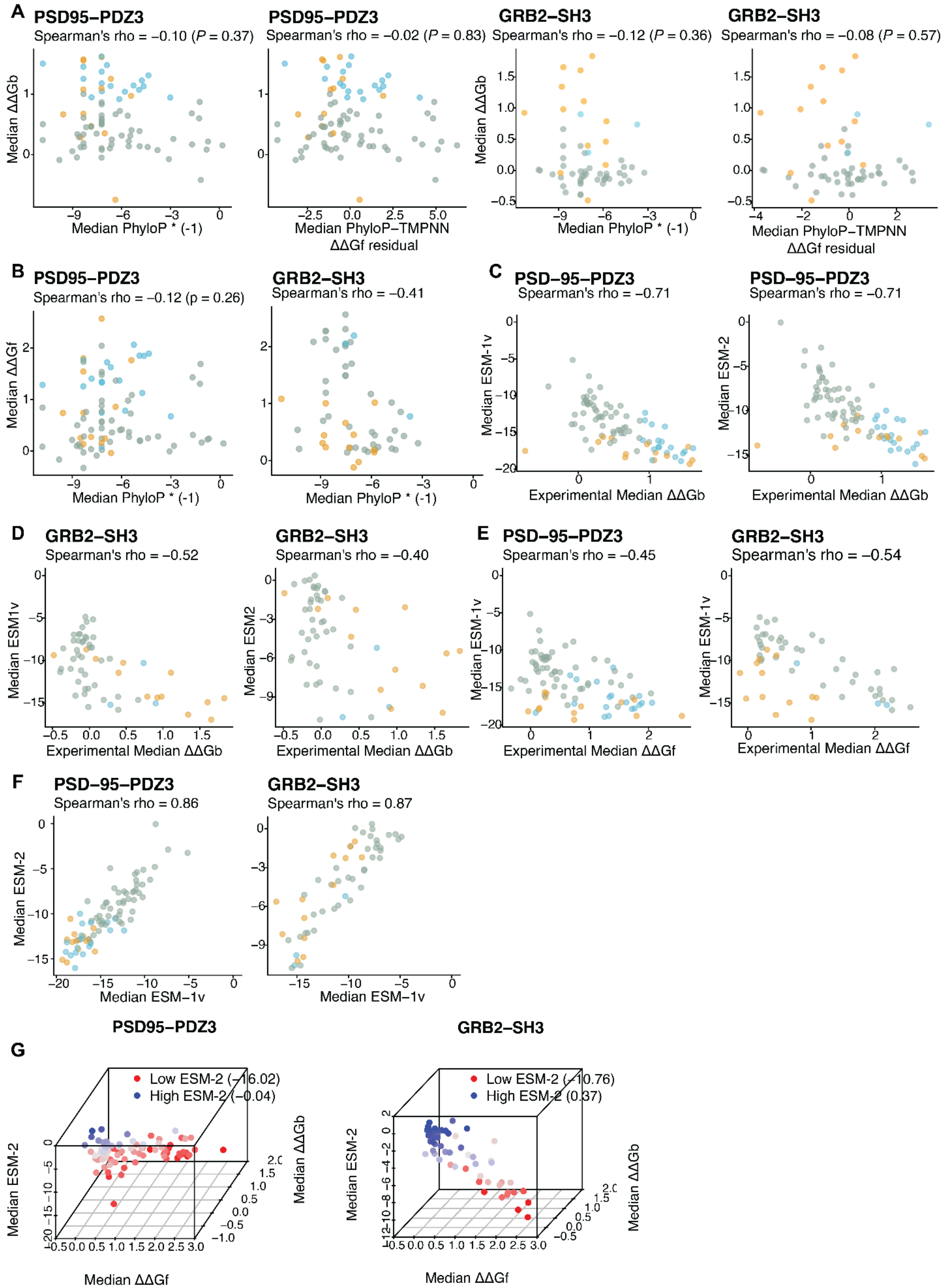
A
PSD95–PDZ3
Spearman's rho = –0.10 (P = 0.37)
Median ΔΔGb
Median PhyloP * (-1)
PSD95–PDZ3
Spearman's rho = –0.02 (P = 0.83)
Median PhyloP–TMPNN ΔΔGf residual
GRB2–SH3
Spearman's rho = –0.12 (P = 0.36)
Median ΔΔGb
Median PhyloP * (-1)
GRB2–SH3
Spearman's rho = –0.08 (P = 0.57)
Median PhyloP–TMPNN ΔΔGf residual
B
PSD95–PDZ3
Spearman's rho = –0.12 (p = 0.26)
Median ΔΔGf
Median PhyloP * (-1)
GRB2–SH3
Spearman's rho = –0.41
Median PhyloP * (-1)
C
PSD–95–PDZ3
Spearman's rho = –0.71
Median ESM-1v
Experimental Median ΔΔGb
PSD–95–PDZ3
Spearman's rho = –0.71
Median ESM-2
Experimental Median ΔΔGb
D
GRB2–SH3
Spearman's rho = –0.52
Median ESM1v
Experimental Median ΔΔGb
GRB2–SH3
Spearman's rho = –0.40
Median ESM2
Experimental Median ΔΔGb
E
PSD–95–PDZ3
Spearman's rho = –0.45
Median ESM-1v
Experimental Median ΔΔGf
GRB2–SH3
Spearman's rho = –0.54
Median ESM-1v
Experimental Median ΔΔGf
F
PSD–95–PDZ3
Spearman's rho = 0.86
Median ESM-2
Median ESM–1v
GRB2–SH3
Spearman's rho = 0.87
Median ESM–1v
G
PSD95–PDZ3
Low ESM-2 (–16.02)
High ESM-2 (–0.04)
Median ESM-2
Median ΔΔGb
Median ΔΔGf
GRB2–SH3
Low ESM-2 (–10.76)
High ESM-2 (0.37)
Median ESM-2
Median ΔΔGb
Median ΔΔGf

Fig. S9: Alternative evolutionary measures and ESM-2 analysis. A. Scatter plots showing the relationship between (left) median PhyloP scores (inverted) and experimentally measured binding free energy ($\Delta\Delta G_b$), and (right) median PhyloP–ThermoMPNN residuals and experimentally measured binding free energy for PSD95-PDZ3 (left) and GRB2-SH3 (right). Spearman correlation coefficients and P values are indicated. Residues are colored by site types: orthosteric (orange), allosteric (blue), other (sage). **B.** Scatter plots showing the relationship between median PhyloP scores (inverted) and experimentally measured folding free energy for PSD95-PDZ3 (left) and GRB2-SH3 (right). **C.** Scatter plots showing the relationship between ESM-1v (left) or ESM-2 (right) scores and experimentally measured binding free energy ($\Delta\Delta G_b$) for PSD95-PDZ3. **D.** Scatter plots show the relationship between ESM-1v (left) or ESM-2 (right) scores and experimentally measured binding free energy ($\Delta\Delta G_b$) for GRB2-SH3. **E.** Scatter plots showing the relationship between ESM-1v scores and experimentally measured $\Delta\Delta G_f$ for PSD95-PDZ3 (left) and GRB2-SH3 (right). **F.** Scatter plots showing the correlation between median ESM-1v and median ESM-2 scores for PSD95-PDZ3 (left) and GRB2-SH3 (right). **G.** 3D scatter plots for PSD95-PDZ3 (left) and GRB2-SH3 (right) mapping median ESM-2 scores (vertical axis) against experimentally measured folding stability ($\Delta\Delta G_f$) and binding free energy ($\Delta\Delta G_b$). Points are colored by ESM-2 score (Low vs High).

Table S1

Proteome-wide predictions of protein stability changes and corresponding ESM-1v scores generated in this study have been deposited in the Zenodo database under accession code 18386427 [https://zenodo.org/records/18386427] as Supplementary Table 1.

Table S2

Protein	Uniprot ID	Function	Name	References
**GCK**	P35557	Enzyme	Glucokinase	Gersing, S., Schulze, T. K., Cagiada, M., Stein, A., Roth, F. P., Lindorff-Larsen, K., & Hartmann-Petersen, R. (2024). Characterizing glucokinase variant mechanisms using a multiplexed abundance assay. Genome biology, 25(1), 98.
**PTEN**	P60484	Enzyme	Phosphatase and tensin homolog	Matreyek, K. A., Starita, L. M., Stephany, J. J., Martin, B., Chiasson, M. A., Gray, V. E., ... & Fowler, D. M. (2018). Multiplex assessment of protein variant abundance by massively parallel sequencing. Nature genetics, 50(6), 874-882.
**VKOR**	Q9BQB6	Enzyme	Vitamin K expoxide reductase	Chiasson, M. A., Rollins, N. J., Stephany, J. J., Sitko, K. A., Matreyek, K. A., Verby, M., ... & Fowler, D. M. (2020). Multiplexed measurement of variant abundance and activity reveals VKOR topology, active site and human variant impact. elife, 9, e58026.
**NUDT15**	Q9NV35	Enzyme	Nucleotide triphosphate diphosphatase	Suiter, C. C., Moriyama, T., Matreyek, K. A., Yang, W., Scaletti, E. R., Nishii, R., ... & Yang, J. J. (2020). Massively parallel variant characterization identifies NUDT15 alleles associated with thiopurine toxicity. Proceedings of the National Academy of Sciences, 117(10), 5394-5401.
**ASPA**	P45381	Enzyme	Aspartoacylase	Grønbæk-Thygesen, M., Voutsinos, V., Johansson, K. E., Schulze, T. K., Cagiada, M., Pedersen, L., ... & Hartmann-Petersen, R. (2024). Deep mutational scanning reveals a correlation between degradation and toxicity of thousands of aspartoacylase variants. Nature Communications, 15(1), 4026.
**TPMT**	P51580	Enzyme	Thiopurine S-methyltransferase	Matreyek, K. A., Starita, L. M., Stephany, J. J., Martin, B., Chiasson, M. A., Gray, V. E., ... & Fowler, D. M. (2018). Multiplex assessment of protein variant abundance by massively parallel sequencing. Nature genetics, 50(6), 874-882.
**CYP2C9**	P11712	Enzyme	Cytochrome P450 enzyme	Amorosi, C. J., Chiasson, M. A., McDonald, M. G., Wong, L. H., Sitko, K. A., Boyle, G., ... & Dunham, M. J. (2021). Massively parallel characterization of CYP2C9 variant enzyme activity and abundance. The American Journal of Human Genetics, 108(9), 1735-1751.
**OCT**	O15245	Transporter	Organic cation transporter 1	Yee, S. W., Macdonald, C. B., Mitrovic, D., Zhou, X., Koleske, M. L., Yang, J., ... & Coyote-Maestas, W. (2024). The full spectrum of SLC22 OCT1 mutations illuminates the bridge between drug transporter biophysics and pharmacogenomics. Molecular cell, 84(10), 1932-1947.
**PRKN**	O60260	Enzyme	E3 ubiquitin-protein ligase parkin	Clausen, L., Okarmus, J., Voutsinos, V., Meyer, M., Lindorff-Larsen, K., & Hartmann-Petersen, R. (2024). PRKN-linked familial Parkinson's disease: cellular and molecular mechanisms of disease-linked variants. Cellular and Molecular Life Sciences, 81(1), 223.

Table. S2. Summary of nine proteins with VAMP-seq abundance measurements.

Table S3

Protein	UniProt ID	Active Site Structure	Active Site Substrate	Active Site Annotation	Allosteric Site Structure	Allosteric Modulator	Allosteric Site Annotation	Type	Slope	Half Distance	t-value	P-value	Adj R2
**GCK**	P35557	1V4S, 3FGU	ANP,BGC, GLC	from 1V4S	1V4S,3ID8	MRK (activator)	literature (Kamata et al., 2004) (Larion & Miller, 2009)	kinase	-0.04	13.88	-9.01	<2e-16	0.16
**PTP1B**	P18031	1PTY	PTR	from 1PTY	1T48,1T49,1T4J	892,BB3,FRJ (inhibitor)	consensus	phosphatase	-0.02	13.99	-4.08	5.97E-05	0.05
**PDK1**	O15530	4RQK, 5LVM	ADE,ATP	consensus	3ORZ,4RQK	2A2 (activator), R1S (inhibitor)	from 4RQK	kinase	-0.05	8.89	-7.84	9.39E-14	0.18
**CHK1**	O14757	2E9N, 5OOP	76A,ANP	consensus	3F9N	38M (inhibitor)	from 3F9N	kinase	-0.04	11.73	-6.9	3.95E-11	0.15
**CASP1**	P29466	2HBQ, 6BZ9	PI (chain C)	consensus	2FQQ	F1G (inhibitor)	from 2FQQ	protease	-0.03	13.31	-4.2	3.74E-05	0.065
**IDH1**	O75874	5YFN, 6ADG, 6B0Z, 6BKZ, 6IO0	FLC,ICT,MG, NAP,NDP	consensus from 6B0Z, 6BKZ, 6IO0	5SUN,6B0Z, 6BKZ,6BL2,6IO0	70Q, AOU, C81, CIT, DWM, DWS (inhibitor)	consensus from 6B0Z, 6BKZ, 6IO0	oxidoreductase	-0.03	12.42	-6.39	5.32E-10	0.1

Table. S3. Summary of six allosteric proteins used for targeted analyses, including protein identifiers, orthosteric and allosteric site definitions, linear regression and distance-dependent decay parameters. Nonlinear least-squares regression was used to fit the distance-dependent decay model. Slope and half-distance values are reported. Linear regression was performed using the `lm()` function in R. P-values for the linear distance coefficient are derived from two-sided t-tests within the linear model framework.

Table S4. PSD-95-PDZ3: linear regression models

	Model Comparison	Res.Df	RSS	Sum of Sq	F	Pr(>F)
whole protein	ESM1v ~ ΔΔGf	1565	21,260			
whole protein	ESM1v ~ ΔΔGf + ΔΔGb	1564	18,199	3,061.20	263.07	< 2.2e-16 ***
non-orthosteric sites	ESM1v ~ ΔΔGf	1323	15,721			
non-orthosteric sites	ESM1v ~ ΔΔGf + ΔΔGb	1322	13,441	2279.3	224.18	< 2.2e-16 ***
orthosteric sites	ESM1v ~ ΔΔGf	240	1845			
orthosteric sites	ESM1v ~ ΔΔGf + ΔΔGb	239	1704.8	140.21	19.657	1.413e-05 ***

Table S5. GRB2-SH3: linear regression model

	Model Comparison	Res.Df	RSS	Sum of Sq	F	Pr(>F)
whole protein	ESM1v ~ ΔΔGf	754	7,968			
whole protein	ESM1v ~ ΔΔGf + ΔΔGb	753	5,795	2,173.30	282.41	< 2.2e-16 ***
non-orthosteric sites	ESM1v ~ ΔΔGf	583	3,265			
non-orthosteric sites	ESM1v ~ ΔΔGf + ΔΔGb	582	3,020	245.48	47.311	1.568e-11 ***
orthosteric sites	ESM1v ~ ΔΔGf	169	1864.3			
orthosteric sites	ESM1v ~ ΔΔGf + ΔΔGb	168	1343	521.27	65.205	1.245e-13 ***

Table S6. PSD-95-PDZ3: linear mixed models

	Model Comparison	npar	AIC	BIC	logLik	-2logLik	Chisq	Df	Pr(>Chisq)
whole protein	ESM1v ~ ΔΔGf + (1 | pos)	4	7575.2	7596.6	-3783.6	7567.2			
whole protein	ESM1v ~ ΔΔGf + ΔΔGb + (1 | pos)	5	7409.7	7436.5	-3699.9	7399.7	167.48	1	< 2.2e-16 ***
non-orthosteric sites	ESM1v ~ ΔΔGf + (1 | pos)	4	6354.3	6375.1	-3173.2	6346.3			
non-orthosteric sites	ESM1v ~ ΔΔGf + ΔΔGb + (1 | pos)	5	6215.8	6241.7	-3102.9	6205.8	140.57	1	< 2.2e-16 ***
orthosteric sites	ESM1v ~ ΔΔGf + (1 | pos)	4	1175.3	1189.2	-583.64	1167.3			
orthosteric sites	ESM1v ~ ΔΔGf + ΔΔGb + (1 | pos)	5	1155.6	1173	-572.78	1145.6	21.705	1	3.179e-06 ***

Table S7. GRB2-SH3: linear mixed models

	Model Comparison	npar	AIC	BIC	logLik	-2logLik	Chisq	Df	Pr(>Chisq)
whole protein	ESM1v ~ ΔΔGf + (1 | pos)	4	3339.9	3358.4	-1666	3331.9			
whole protein	ESM1v ~ ΔΔGf + ΔΔGb + (1 | pos)	5	3304.1	3327.2	-1647	3294.1	37.858	1	7.609e-10 ***
non-orthosteric sites	ESM1v ~ ΔΔGf + (1 | pos)	4	2461.3	2478.8	-1226.7	2453.3			
non-orthosteric sites	ESM1v ~ ΔΔGf + ΔΔGb + (1 | pos)	5	2434.5	2456.4	-1212.3	2424.5	28.795	1	8.047e-08 ***
orthosteric sites	ESM1v ~ ΔΔGf + (1 | pos)	4	810.36	822.92	-401.18	802.36			
orthosteric sites	ESM1v ~ ΔΔGf + ΔΔGb + (1 | pos)	5	807.81	823.52	-398.91	797.81	4.5424	1	0.03307 *

Table. S4. Ordinary linear regression results for PSD-95-PDZ3. Models were fitted using the `lm()` function in R, regressing ESM-1v scores on folding $\Delta\Delta G_f$, binding $\Delta\Delta G_b$, or both, stratified by mutation class (all, non-orthosteric, orthosteric). Nested models were compared using ANOVA F-tests. *P*-values are two-sided. Significance codes: *** $P < 0.001$, ** $P < 0.01$, * $P < 0.05$. **Table. S5.** Ordinary linear regression results for GRB2-SH3. Models were fitted using the `lm()` function in R, regressing ESM-1v scores on folding $\Delta\Delta G_f$, binding $\Delta\Delta G_b$, or both, stratified by mutation class (all, non-orthosteric, orthosteric). Nested models were compared using ANOVA F-tests. *P*-values are two-sided. **Table. S6.** Linear mixed-effects model results for PSD-95-PDZ3. Models were fitted using the `lmer()` function in R, regressing ESM-1v scores on folding $\Delta\Delta G_f$, binding $\Delta\Delta G_b$, or both, with per-position random intercepts. Models were stratified by mutation class (all, non-orthosteric, orthosteric). Nested models were compared using likelihood-ratio tests (χ^2 test via ANOVA). P-values are two-sided. **Table. S7.** Linear mixed-effects model results for GRB2-SH3. Models were fitted using the `lmer()` function in R, regressing ESM-1v scores on folding $\Delta\Delta G_f$, binding $\Delta\Delta G_b$, or both, with per-position random intercepts. Models were stratified by mutation class (all, non-orthosteric, orthosteric). Nested models were compared using likelihood-ratio tests (χ^2 test via ANOVA). P-values are two-sided.
