# Supplementary material for: Allostery is a widespread cause of loss-of-function variant pathogenicity
Source: Nat Commun. 2026 Jun 22;17:7841. doi: 10.1038/s41467-026-74517-8 (PMC13439481; doi:10.1038/s41467-026-74517-8)
Supplement: Supplementary file 2 [file 41467_2026_74517_MOESM2_ESM.pdf]

nature portfolio

Corresponding author(s): Ben Lehner

Last updated by author(s): Apr 28, 2026

Reporting Summary

Nature Portfolio wishes to improve the reproducibility of the work that we publish. This form provides structure for consistency and transparency in reporting. For further information on Nature Portfolio policies, see our Editorial Policies and the Editorial Policy Checklist.

Statistics

For all statistical analyses, confirm that the following items are present in the figure legend, table legend, main text, or Methods section.

n/a	Confirmed	
☐	☒	The exact sample size (*n*) for each experimental group/condition, given as a discrete number and unit of measurement
☐	☒	A statement on whether measurements were taken from distinct samples or whether the same sample was measured repeatedly
☐	☒	The statistical test(s) used AND whether they are one- or two-sided<br>*Only common tests should be described solely by name; describe more complex techniques in the Methods section.*
☒	☐	A description of all covariates tested
☒	☐	A description of any assumptions or corrections, such as tests of normality and adjustment for multiple comparisons
☐	☒	A full description of the statistical parameters including central tendency (e.g. means) or other basic estimates (e.g. regression coefficient) AND variation (e.g. standard deviation) or associated estimates of uncertainty (e.g. confidence intervals)
☐	☒	For null hypothesis testing, the test statistic (e.g. *F*, *t*, *r*) with confidence intervals, effect sizes, degrees of freedom and *P* value noted<br>*Give P values as exact values whenever suitable.*
☒	☐	For Bayesian analysis, information on the choice of priors and Markov chain Monte Carlo settings
☒	☐	For hierarchical and complex designs, identification of the appropriate level for tests and full reporting of outcomes
☐	☒	Estimates of effect sizes (e.g. Cohen's *d*, Pearson's *r*), indicating how they were calculated

Our web collection on statistics for biologists contains articles on many of the points above.

Software and code

Policy information about availability of computer code

Data collection	All datasets used in this study are open-source and publicly available. Large-scale experimental measurements of protein abundance changes across human domains, including datasets from the Mega-scale and Domainome studies, are accessible via their respective supplementary materials. VAMP-seq abundance data are available from MaveDB[https://doi.org/10.1186/s13059-019-1845-6] and ProteinGym [https://doi.org/10.1101/2023.12.07.570727]91,116. Experimental ΔΔG values for PSD95-PDZ3 and GRB2-SH3 domains, and experimental measurements of abundance and activity-based fitness for PTEN and GCK, and SGE scores, are available through the original studies cited in the references.
Data analysis	All code used for analysis is at https://github.com/lehner-lab/allostery_pathogenicity. Softwares used include R version 4.5.0 and ChimeraX version 1.6.1. Plots were generated using the ggplot2 package version 4.0.3.

For manuscripts utilizing custom algorithms or software that are central to the research but not yet described in published literature, software must be made available to editors and reviewers. We strongly encourage code deposition in a community repository (e.g. GitHub). See the Nature Portfolio guidelines for submitting code & software for further information.

Data

Policy information about availability of data

All manuscripts must include a data availability statement. This statement should provide the following information, where applicable:

- Accession codes, unique identifiers, or web links for publicly available datasets
- A description of any restrictions on data availability
- For clinical datasets or third party data, please ensure that the statement adheres to our policy

Proteome-wide predictions of protein stability changes and corresponding ESM-1v scores generated in this study have been deposited in the Zenodo database under accession code 18386427[https://zenodo.org/records/18386427] as Supplementary Table 1. Raw ThermoMPNN predictions of protein stability changes generated in this study are available at Zenodo under accession code 18381534[https://zenodo.org/records/18381534].

Large-scale experimental measurements of protein abundance changes across human domains, including datasets from the Mega-scale and Domainome studies, are accessible via their respective supplementary materials. VAMP-seq abundance data are available from MaveDB[https://doi.org/10.1186/s13059-019-1845-6] and ProteinGym [https://doi.org/10.1101/2023.12.07.570727]91,116. Experimental ΔΔG values for PSD95-PDZ3 and GRB2-SH3 domains, and experimental measurements of abundance and activity-based fitness for PTEN and GCK, and SGE scores, are available through the original studies cited in the references.

PDB codes include 1D5R[https://doi.org/10.2210/pdb1D5R/pdb], 1V4S[https://doi.org/10.2210/pdb1V4S/pdb], 1T29[https://doi.org/10.2210/pdb1T29/pdb], 1JM7 [https://doi.org/10.2210/pdb1JM7/pdb], 1BE9[https://doi.org/10.2210/pdb1BE9/pdb], 2VWF[https://doi.org/10.2210/pdb2VWF/pdb], 4RQK[https://doi.org/10.2210/pdb4RQK/pdb], 1PTY[https://doi.org/10.2210/pdb1PTY/pdb], 2HBQ[https://doi.org/10.2210/pdb1HBQ/pdb], 2E9N[https://doi.org/10.2210/pdb2E9N/pdb], 6IO0[https://doi.org/10.2210/pdb6IO0/pdb]. Source data are provided with this paper.

Research involving human participants, their data, or biological material

Policy information about studies with human participants or human data. See also policy information about sex, gender (identity/presentation), and sexual orientation and race, ethnicity and racism.

Reporting on sex and gender	Not applicable
Reporting on race, ethnicity, or other socially relevant groupings	Not applicable
Population characteristics	Not applicable
Recruitment	Not applicable
Ethics oversight	Not applicable

Note that full information on the approval of the study protocol must also be provided in the manuscript.

Field-specific reporting

Please select the one below that is the best fit for your research. If you are not sure, read the appropriate sections before making your selection.

☒ Life sciences ☐ Behavioural & social sciences ☐ Ecological, evolutionary & environmental sciences

For a reference copy of the document with all sections, see nature.com/documents/nr-reporting-summary-flat.pdf

Life sciences study design

All studies must disclose on these points even when the disclosure is negative.

Sample size	Not applicable
Data exclusions	Not applicable
Replication	Not applicable
Randomization	Not applicable
Blinding	Not applicable

Reporting for specific materials, systems and methods

We require information from authors about some types of materials, experimental systems and methods used in many studies. Here, indicate whether each material, system or method listed is relevant to your study. If you are not sure if a list item applies to your research, read the appropriate section before selecting a response.

Materials & experimental systems

n/a	Involved in the study
☒	☐ Antibodies
☒	☐ Eukaryotic cell lines
☒	☐ Palaeontology and archaeology
☒	☐ Animals and other organisms
☒	☐ Clinical data
☒	☐ Dual use research of concern
☒	☐ Plants

Methods

n/a	Involved in the study
☒	☐ ChIP-seq
☒	☐ Flow cytometry
☒	☐ MRI-based neuroimaging

Plants

Seed stocks	Not applicable
Novel plant genotypes	Not applicable
Authentication	Not applicable
