# Supplementary material for: Allostery is a widespread cause of loss-of-function variant pathogenicity
Source: Nat Commun. 2026 Jun 22;17:7841. doi: 10.1038/s41467-026-74517-8 (PMC13439481; doi:10.1038/s41467-026-74517-8)
Supplement: Supplementary file 3 [file 41467_2026_74517_MOESM3_ESM.pdf]

nature portfolio

Peer Review File

Allostery is a widespread cause of loss-of-function variant pathogenicity

Corresponding Author: Professor Ben Lehner

This file contains all reviewer reports in order by version, followed by all author rebuttals in order by version.

Attachments originally included by the reviewers as part of their assessment can be found at the end of this file.

Version 0:

Reviewer comments:

Reviewer #1

(Remarks to the Author)
Review of Liao et al. Allostery is a widespread cause of loss-of-function variant pathogenicity.
Liao and Lehner hypothesize that variants that affect allostery could be a source of potentially pathogenic variation. They devise in silico analyses to explore this hypothesis. They determine that stability, as measured by experiments or ThermoMPNN, cannot completely account for LLM-based evolutionary models of pathogenicity. This result reinforces the original findings from the publications describing the experimental data that multiplexed experiments that measure protein abundance can only identify a fraction of pathogenic control variants. They then regress out stability to compare ESM1v to distance to active site and determine that estimates of pathogenicity decays with distance. Comparison to DMS where both stability and activity have been measured shows that variants at allosteric sites have greater than predicted functional effects. The connection between allosteric residues and pathogenic variation is perhaps not surprising given basic principles of protein function, but Liao and Lehner have usefully formalized and validated this relationship with this study.
Can you replicate Fig1 and Fig2 results with AlphaMissense? Or EVE? Do you even need a state of the art predictor? Would PhyloP do the job?
ESM1v is not a perfect predictor of pathogenicity. What part of the missing function beyond stability is due to poor prediction and specifically over-prediction of pathogenicity? Maybe compare ESM1V to comprehensive function datasets like SGE (which generally have near perfect ROC-AUC for path/benign variants, unlike ESM1v) to get an estimate of the Δ between SGE and ESM1v to understand how much of the pathogenicity prediction is missing?
I would not call the PTEN disease classifier from Mighell 2020 “established criteria”. It is quite difficult to disentangle variants that cause PTEN phenotypes without curating the clinical literature. Between that and the small N, I think Fig 4E could be misleading. Maybe just don’t go there without clinically curated cases?
The GCK dataset has imputed scores for missing variants. How was that handled? We’re the imputed scores included, or did you use only the real functional measurements?

Minor
Page and line numbers would have been nice!
ClinVar isn’t highly curated. It’s kind of notorious for having deprecated stuff in there. In fact, you could add supplemental figures using only 3+-star variants which are highly curated to further your point.
Fig 4G has misplaced pipes on the Y axis labels

Lea Starita
University of Washington

(Remarks on code availability)

Reviewer #2

(Remarks to the Author)
Summary
In this article, Liao and Lehner note that many pathogenic mutations are likely to occur at allosteric residues. Using predictions from two machine learning models trained to predict evolutionary conservation (ESM-v1) and thermostability (ThermoMPNN), the authors posit that residues with mutational effects not captured by either of these methods (high stability and high pathogenicity or low stability and low pathogenicity) likely represent allosteric mutations. Using published datasets, the authors repeat similar analyses across many proteins and datasets finding that pathogenic mutations are enriched for positions that are likely allosteric—the positions that are distal to an orthosteric site with a high, unexplained mutational effect.

This paper makes an interesting observation, noting that two deep learning models are imperfect oracles of protein function. However, there is very little discussion of prior work on allostery, co-evolution, or the limitations of these models and the tasks they were trained to perform. Without this discussion and comparison to conventional methods, the findings are not well situated in the existing literature to help readers draw concrete conclusions or build upon this research. These comments are provided to help improve the paper.

Major Comments:

There needs to be more discussion of allostery and deep learning in the introduction. The introduction and discussion focus mainly on pathogenic variants, while the text and results rely heavily on understanding how these models were trained and on current conceptual models for allostery.
The analysis in the paper is centered around the residual deviance when accounting for ESM-1v and ThermoMPNN predictions; however, it is unclear to what extent each model contributes. A figure displaying the deviance as a function of each individual predictor would be helpful in assessing the utility of using both predictors.
Are there proteins where ThermoMPNN fits the data perfectly – small single domain proteins with small cores – and ESM1v still has residual deviance? Are there examples of the opposite?
Since the publication of ESM-1b, multiple ESM models (ESM-2, ESM-3, ESM-C) have been published, in addition to other protein language models. The framework for ESM-1v calculations—the likelihood ratio between the wild-type and mutated amino acid at a masked position—can be accomplished with L forward passes of a model for a protein with length L. The authors should repeat their analysis for a subset of the proteins using more modern language models. Are models trained with more parameters and data better able to capture these relationships? I worry that newer language models trained with more data may actually capture these allosteric and orthosteric relationships, discounting the need for ThermoMPNN.
Allostery has long been posited to involve co-evolving residues spread through the core of a protein (DOI: 10.1016/j.cell.2009.07.038). For residues that are poorly explained by protein language models, could this be due to a lack of MSA depth at those positions? Do conventional methods for the detection of co-evolution such as direct coupling analysis (DOI: 10.7554/eLife.09248.001) correctly identify positions where there is sufficient MSA depth?
ThermoMPNN has known biases, including the prediction of hydrophobic residues at surface-exposed and core positions. How do these biases map onto the set of clinical variants?
How many of the pathogenic variants analyzed have poor (below 70) pLDDT scores in AlphaFold2 and may thus have poor sequence recovery by ProteinMPNN?
There appear to be many green points (e.g., Figure 2D) that have high deviance and are not pathogenic. How do the authors think about these positions?
In Figure 1C, we see that immune signaling receptors have an increased, albeit small, correlation between protein stability and pathogenicity. How do hypermutant proteins deviate in terms of distance dependence and co-evolution of core residues using biased models

Minor Comments:
The figures need simplification. It is hard to glean the main point of a figure from looking at and the same information is replicated multiple times both within and across figures.
It is hard to see the ligands displayed in yellow (Figure 2).
Typo in Figure 2: “predicted variant structural stability.”
Figure 1G should more clearly distinguish the ClinVar variants that are core vs. non-core

(Remarks on code availability)

Version 1:

Reviewer comments:

Reviewer #1

(Remarks to the Author)

This added sentence in the introduction "Most pathogenic LOF variants are missense mutations" is wildly untrue. Please remember that nonsense mediated decay and splicing are a thing and damaging the RNA is epistatic to and potentially even

more damaging than missense variation that may be buffered by chaparones in cells. For single nucleotide variants labelled pathogenic or likely pathogenic in ClinVar (179,885), missense (68,859) only slightly outnumber nonsense (67,098), even though missense variants are much easier to make with a single base change. Nonsense (67,098) + splice (38,717) variants greatly outnumber missense variants (68,859). And even then, I'd bet that many of the pathogenic missense variants are actually causing splice defects if you dig into the details. In fact, many clinical geneticists don't even believe missense LOF variants causing protein-effects can be pathogenic at all for some genes (e.g. the new VCEP rules for PALB2 variant classification). Delete this sentence.

Otherwise the updated version has addressed my concerns.

(Remarks on code availability)

Reviewer #2

(Remarks to the Author)
The paper has been improved significantly from the first submission and my reviews were addressed. I have no additional comments or concerns.

(Remarks on code availability)

Open Access This Peer Review File is licensed under a Creative Commons Attribution 4.0 International License, which permits use, sharing, adaptation, distribution and reproduction in any medium or format, as long as you give appropriate credit to the original author(s) and the source, provide a link to the Creative Commons license, and indicate if changes were made.
In cases where reviewers are anonymous, credit should be given to 'Anonymous Referee' and the source.
The images or other third party material in this Peer Review File are included in the article’s Creative Commons license, unless indicated otherwise in a credit line to the material. If material is not included in the article’s Creative Commons license and your intended use is not permitted by statutory regulation or exceeds the permitted use, you will need to obtain permission directly from the copyright holder.
To view a copy of this license, visit https://creativecommons.org/licenses/by/4.0/

We thank the reviewers for their insightful feedback and constructive criticism. We are grateful for the suggestions to validate our findings with additional predictors (AlphaMissense, ESM-2), higher-quality clinical data (3-star ClinVar variants), and orthogonal ground-truth datasets (SGE). These additional analyses have significantly strengthened the manuscript.

In the revised manuscript, we have made the following updates:

1. **Validation with AlphaMissense & ESM-2:** We replicated our key findings using AlphaMissense and ESM-2, demonstrating that the detection of allosteric decay is robust to the choice of state-of-the-art predictor.
2. **Benchmarking against Experimental Ground Truth:** We analyzed BRCA1, RAD51C, and BAP1(Saturation Genome Editing (SGE) data to show that the "missing function" (residuals) in our model represents genuine biological signal rather than model noise.
3. **Strict Data Curation:** We replicated our analyses using only high-confidence (3+ star) ClinVar variants and filtering for high-confidence structural regions (pLDDT > 70).
4. **Expanded Introduction:** We have updated the Introduction to better contextualize our work within the history of co-evolutionary methods and to explicitly discuss the limitations of the deep learning models used.
5. **Structural Reorganization:** We have cleaned up the figures for clarity and rearranged the order of the Results section and Figures to create a clearer narrative.

Reviewer #1 (Remarks to the Author):

Review of Liao et al. Allostery is a widespread cause of loss-of-function variant pathogenicity. Liao and Lehner hypothesize that variants that affect allostery could be a source of potentially pathogenic variation. They devise in silico analyses to explore this hypothesis. They determine that stability, as measured by experiments or ThermoMPNN, cannot completely account for LLM-based evolutionary models of pathogenicity. This result reinforces the original findings from the publications describing the experimental data that multiplexed experiments that measure protein abundance can only identify a fraction of pathogenic control variants. They then regress out stability to compare ESM1v to distance to active site and determine that estimates of pathogenicity decays with distance. Comparison to DMS where both stability and activity have been measured shows that variants at allosteric sites have greater than predicted functional effects. The connection between allosteric residues and pathogenic variation is perhaps not surprising given basic principles of protein function, but Liao and Lehner have usefully formalized and validated this relationship with this study.

Can you replicate Fig1 and Fig2 results with AlphaMissense? Or EVE?
We thank the reviewer for this suggestion. To address this, we replicated the analyses in Figure 1 and 2 using AlphaMissense.

We found the results to be highly concordant with those obtained using ESM-1v. Specifically:

1. **Replication of Figure 1:** Predicted folding stability explains only a fraction of the AlphaMissense logit scores. The median R^2 between AlphaMissense logits and predicted folding stability is 0.275 (all variants) and 0.18 (core variants), which is comparable to the variances observed with ESM-1v (as compared to 0.224 and 0.158 with ESM-1v, **Supplemental Figure 1C**).
2. **Replication of Figure 2:** We continue to observe a clear distance-dependent decay of functional residuals (AlphaMissense logits-ThermoMPNN ΔΔGf) away from the active site **(Supplemental Figure 8F)**. The residuals derived from AlphaMissense correlate strongly with those derived from ESM-1v. We observed high Spearman correlations across proteins (Spearman's ρ = 0.60 (PDK1), 0.78 (GCK), 0.71 (PTP1B), 0.66 (CHK1), 0.65 (CASP1), 0.79 (IDH)).

These results, now included in **Supplemental Figure 1C, F and Supplemental Figure 8F**, confirm that our findings are robust to the choice of pathogenicity predictor.

Do you even need a state of the art predictor? Would PhyloP do the job?
We investigated whether simpler evolutionary conservation metrics, such as PhyloP, could capture these effects. Our analysis confirms that a state-of-the-art protein language model is essential to resolve these signals.

When we replicated our analysis using PhyloP, we found that it correlates more strongly with folding stability (ΔΔGf) than with experimental binding free energies. Consequently, residuals derived from PhyloP failed to discriminate orthosteric and allosteric sites from background residues. In contrast, ESM-1v correlates much better with binding free energy (Table 1).

We have now included these results in **Supplemental Figure 9A-E**. This distinction is consistent with recent work suggesting that traditional conservation metrics are dominated by stability constraints, whereas modern language models better capture specific functional effects(Cagiada et al. 2021; Livesey and Marsh 2023).

	PSD-95-PDZ3 ΔΔGf	PSD-95-PDZ3 ΔΔGb	GRB2-SH3 ΔΔGf	GRB2-SH3 ΔΔGb
PhyloP	ρ = -0.12, *P* > 0.05	ρ = -0.10, *P* > 0.05	ρ = -0.41, *P* = 0.002	ρ = -0.12, *P* > 0.05
ESM-1v	ρ = -0.45, *P* < 0.001	ρ = -0.71, *P* < 0.001	ρ = -0.54, *P* < 0.001	ρ = -0.52, *P* < 0.001

Table 1. Comparison of Spearman correlations between PhyloP versus ESM-1v and ΔΔGb.

ESM1v is not a perfect predictor of pathogenicity. What part of the missing function beyond stability is due to poor prediction and specifically over-prediction of pathogenicity? Maybe compare ESM1V to comprehensive function datasets like SGE (which generally have near

perfect ROC-AUC for path/benign variants, unlike ESM1v) to get an estimate of the ∆ between SGE and ESM1v to understand how much of the pathogenicity prediction is missing?

This is an excellent suggestion. To differentiate between "true biological signal" and "prediction noise," we have added a new section to the Results and a new **Figure 3** benchmarking our framework against high-quality Saturation Genome Editing (SGE) datasets for BRCA1, RAD51C, and BAP1(Findlay et al. 2018; Olvera-León et al. 2024; Waters et al. 2024).

Because SGE scores provide an experimental 'ground truth' for cellular function (at least in one cell type), they allow us to estimate how much of the ESM-1v prediction is 'missing' (error) versus how much is tracking a stability-independent phenotype (biology).

To make the computational and experimental metrics directly comparable, we applied rank-based normalization to scale both the SGE scores and ESM-1v predictions onto a shared 0–1 axis, where 1 represents maximum pathogenicity. We then decomposed the variance into two defined components:

1. **Biological Signal:** the difference between evolutionary fitness and biophysical stability (ESM-1v–ThermoMPNN ΔΔGf $residual_{norm}$)
2. **Predictive Noise:** the difference between the experimental ground truth and the evolutionary prediction($\Delta = SGE_{norm} – ESM\text{-}1v_{norm}$)

Our analysis (**Figure 3, Supplemental Figure 3&4**) reveals that the magnitude of the "Biological Signal" is consistently larger than the "Predictive Noise," indicating that the residuals are driven primarily by real phenotypic effects rather than model error. Interestingly, while the "Biological Signal" exhibits a clear distance-dependent decay from orthosteric sites/binding interfaces as shown in the initial submission, the "Predictive Noise" is spatially uniform across distance.

We have included the full text describing these findings in the new Results section titled 'Clinically pathogenic variants in SGE data' (line 204-246) and detailed the normalization procedure in the Methods (line 439-444).

I would not call the PTEN disease classifier from Mighell 2020 “established criteria”. It is quite difficult to disentangle variants that cause PTEN phenotypes witlhout curating the clinical literature. Between that and the small N, I think Fig 4E could be misleading. Maybe just don’t go there without clinically curated cases?

Following the reviewer's suggestion, we have removed the phenotypic stratification analysis (formerly Figure 4E) from the manuscript to avoid potential misinterpretation.

The GCK dataset has imputed scores for missing variants. How was that handled? We’re the imputed scores included, or did you use only the real functional measurements?

We used only the real functional measurements, not imputed scores. We have updated the Methods section to explicitly state this criterion (line 426-427).

Minor
Page and line numbers would have been nice!
Page and line numbers have now been added.

ClinVar isn't highly curated. It's kind of notorious for having deprecated stuff in there. In fact, you could add supplemental figures using only 3+-star variants which are highly curated to further your point.
We appreciate this suggestion and have repeated our proteome-wide analysis using only variants with a review status of 3 stars or higher.

Analyzing 1,974 3-star variants across 57 proteins, we found the results to be consistent with the full dataset (**Supplemental Figure 1A**)

1. Among all 3-star pathogenic variants, 60.3% are destabilizing (compared to 48.4% in the full dataset).
2. For core 3-star variants, 75.9% are destabilizing (compared to 67.2% in the full dataset).

Fig 4G has misplaced pipes on the Y axis labels
Figure 4G has been updated and is now Figure 3E.

Lea Starita
University of Washington

Reviewer #2 (Remarks to the Author):

Summary
In this article, Liao and Lehner note that many pathogenic mutations are likely to occur at allosteric residues. Using predictions from two machine learning models trained to predict evolutionary conservation (ESM-v1) and thermostability (ThermoMPNN), the authors posit that residues with mutational effects not captured by either of these methods (high stability and high pathogenicity or low stability and low pathogenicity) likely represent allosteric mutations. Using published datasets, the authors repeat similar analyses across many proteins and datasets finding that pathogenic mutations are enriched for positions that are likely allosteric—the positions that are distal to an orthosteric site with a high, unexplained mutational effect.

This paper makes an interesting observation, noting that two deep learning models are imperfect oracles of protein function. However, there is very little discussion of prior work on allostery, co-evolution, or the limitations of these models and the tasks they were trained to perform. Without this discussion and comparison to conventional methods, the findings are not well situated in the existing literature to help readers draw concrete conclusions or build upon this research. These comments are provided to help improve the paper.
Major Comments:

There needs to be more discussion of allostery and deep learning in the introduction. The introduction and discussion focus mainly on pathogenic variants, while the text and results rely

heavily on understanding how these models were trained and on current conceptual models for allostery.

We thank the reviewer for this important suggestion and agree that situating our work within the rich history of co-evolutionary analysis is essential. We have significantly expanded the Introduction to address this.

- We now discuss how our approach builds upon traditional statistical methods such as Statistical Coupling Analysis (SCA) and Direct Coupling Analysis (DCA).
- We explicitly contrast the training objectives of Protein Language Models (ESM-1v), which capture "total evolutionary fitness" (including function and co-evolution), versus models trained to predict specific biophysical properties (ThermoMPNN). This context clarifies why comparing these models allows us to isolate allosteric effects.

The analysis in the paper is centered around the residual deviance when accounting for ESM-1v and ThermoMPNN predictions; however, it is unclear to what extent each model contributes. A figure displaying the deviance as a function of each individual predictor would be helpful in assessing the utility of using both predictors. Are there proteins where ThermoMPNN fits the data perfectly – small single domain proteins with small cores – and ESM1v still has residual deviance? Are there examples of the opposite?

We thank the reviewer for this suggestion. To explicitly visualize the individual contribution of each model, we have added **Supplemental Figure 8C-D**, which plots ESM-1v and ThermoMPNN predictions separately against experimental stability and binding data for two small protein domains: PSD95-PDZ3 and GRB2-SH3.

Our analysis shows:

1. As the reviewer hypothesized, ThermoMPNN fits the experimental stability data well for these small protein domains (Spearman's $\rho = 0.62$ for PDZ3; $\rho = 0.76$ for SH3). However, it fails to discriminate residues that disrupt binding from wild-type; experimentally validated residues important for binding (red points) are indistinguishable from other positions.
2. ESM-1v captures both, creating "functional outliers": ESM-1v shows a moderate correlation with folding free energy ($\rho = -0.45$ and -0.54, respectively). Crucially, however, residues important for binding appear as distinct outliers: they are predicted to be highly deleterious by ESM-1v despite having small measured changes in folding free energy.

This side-by-side comparison demonstrates why the residual framework is necessary: ThermoMPNN provides the stability baseline required to identify which low-fitness variants in ESM-1v are not explained by folding constraints, thereby isolating the functional signal.

Since the publication of ESM-1b, multiple ESM models (ESM-2, ESM-3, ESM-C) have been published, in addition to other protein language models. The framework for ESM-1v calculations—the likelihood ratio between the wild-type and mutated amino acid at a masked position—can be accomplished with L forward passes of a model for a protein with length L. The authors should repeat their analysis for a subset of the proteins using more modern language

models. Are models trained with more parameters and data better able to capture these relationships? I worry that newer language models trained with more data may actually capture these allosteric and orthosteric relationships, discounting the need for ThermoMPNN.
We thank the reviewer for this suggestion. We tested whether a more modern, larger-parameter language model (ESM-2, 3B parameters) could capture these functional relationships more explicitly, potentially rendering the "subtraction" of ThermoMPNN unnecessary.

We repeated our analysis using ESM-2 for the PSD95-PDZ3 and GRB2-SH3 datasets where experimental binding and stability measurements are available.

1. **ESM-2 performs similarly to ESM-1v**: We found that ESM-2 and ESM-1v predictions are highly correlated (**Supplemental Figure 9F**, Spearman's ρ = 0.86 and 0.87 respectively). Furthermore, ESM-2 performs similarly to ESM-1v against experimental benchmarks: in PSD95-PDZ3, both models achieve a Spearman's ρ of -0.71, and in GRB2-SH3, performance is comparable (ρ = -0.52 for ESM-1v vs. -0.40 for ESM-2, **Supplemental Figure 9C,D**).
2. **ThermoMPNN is still required to deconvolute mechanism**: The reviewer hypothesized that newer models might distinguish allosteric/orthosteric effects well enough to discount the need for ThermoMPNN. To test this, we generated 3D scatter plots (**Supplemental Figure 9G**) mapping ESM-2 scores against both experimental stability and binding. We observed that the variants with the lowest (predicted to be the most deleterious) ESM-2 scores fall into two distinct biophysical categories: those with large stability defects (large ΔΔGb) and those with large binding defects (large ΔΔGb). Because the language model assigns "poor scores" to both categories indiscriminately (capturing global fitness), the stability signal is still conflated with the functional signal. Therefore, ThermoMPNN remains essential: it provides the independent stability baseline required to "regress out" the ΔΔGf component, thereby isolating the specific functional/allosteric variants that ESM-1v or ESM-2 flags as deleterious but cannot mechanistically classify.

Allostery has long been posited to involve co-evolving residues spread through the core of a protein (DOI: 10.1016/j.cell.2009.07.038). For residues that are poorly explained by protein language models, could this be due to a lack of MSA depth at those positions?
We investigated whether alignment depth confounds our analysis by calculating the effective number of sequences N_{eff} per position. We defined global N_{eff} by clustering sequences at 80% identity and derived positional N_{eff} by scaling this global value by the non-gap coverage (1 - gap frequency) at each site.

We found that the correlation between positional N_{eff} and the ESM-1v–ThermoMPNN ΔΔGf residuals is weak and statistically insignificant (**Supplemental Figure 8A**, PSD-95-PDZ3: Spearman's ρ =-0.16, $P > 0.05$; GRB2-SH3: Spearman's ρ = -0.19, $P > 0.05$).

Do conventional methods for the detection of co-evolution such as direct coupling analysis (DOI: 10.7554/eLife.09248.001) correctly identify positions where there is sufficient MSA depth?

In line with this suggestion, we calculated the maximum DCA coupling strength between each residue and any residue in the orthosteric binding surfaces for PSD95-PDZ3 and GRB2-SH3.

As shown in **Supplemental Figure 8B**, we found that this metric exhibits weak and statistically insignificant correlations with binding free energy (Spearman's ρ = -0.07 for PSD-95-PDZ3; ρ = 0.32 for GRB2-SH3; $P > 0.05$). However, we recognize that taking the maximum pairwise coupling is a relatively naive way of utilizing DCA. It is entirely possible that more sophisticated statistical approaches could successfully identify these functional networks.

ThermoMPNN has known biases, including the prediction of hydrophobic residues at surface-exposed and core positions. How do these biases map onto the set of clinical variants? How many of the pathogenic variants analyzed have poor (below 70) pLDDT scores in AlphaFold2 and may thus have poor sequence recovery by ProteinMPNN?

We thank the reviewer for this important consideration. We found that 50.5% of benign variants and 13.7% of pathogenic variants fall in regions with low AlphaFold confidence (pLDDT ≤ 70).

To ensure our conclusions were not driven by poor sequence recovery in these regions, we repeated our analysis using only high-confidence residues (pLDDT > 70). The results remained consistent with the full dataset, as shown in **Supplemental Figure 1B**. Specifically, among high-confidence pathogenic variants, 55.2% are destabilizing (compared to 48.4% in the full dataset), and for core variants, 67.5% are destabilizing (compared to 67.2% in the full dataset).

There appear to be many green points (e.g., Figure 2D) that have high deviance and are not pathogenic. How do the authors think about these positions?
We thank the reviewer for this observation. We believe the new analyses of Saturation Genome Editing (SGE) data presented in our response to Reviewer 1 (**Figure 3, Supplemental Figure 3&4**) is particularly relevant here. This analysis showed that the biological signal consistently exceeds model error and follows a spatially coherent distance-dependent decay, whereas predictive noise is spatially uniform. We have now added a sentence to the Discussion section (line 349-352) to clarify that these high-deviance 'green' points likely represent unannotated variants, sub-clinical functional loss that does not manifest as disease, or residual predictive noise.

In Figure 1C, we see that immune signaling receptors have an increased, albeit small, correlation between protein stability and pathogenicity. How do hypermutant proteins deviate in terms of distance dependence and co-evolution of core residues using biased models
In line with this suggestion, we performed additional analyses on three representative immune receptors where ESM-1v scores correlate well (Spearman's ρ = -0.59, -0.53, and -0.59, respectively) with predicted stability (ThermoMPNN ΔΔGf): immunoglobulin kappa variable 1D-16, T cell receptor alpha variable 18, and immunoglobulin kappa variable 3D-7. We quantified the decay of the absolute median ESM-1v–ThermoMPNN ΔΔGf residual relative to the minimal distance from the Complementarity Determining Regions (CDRs). We found that even in these hypermutant contexts, a clear distance-dependent decay from the CDRs is present, consistent

with the patterns observed in non-hypermutant proteins. These results are now presented in **Supplemental Figure 8E**.

Minor Comments:

The figures need simplification. It is hard to glean the main point of a figure from looking at and the same information is replicated multiple times both within and across figures.

We thank the reviewer for this constructive criticism. We agree that some figures were overly dense and that the narrative flow could be improved. In the revised manuscript, we have undertaken a significant simplification and reorganization of the main figures to improve clarity and remove redundancy:

- **Removed Redundant Panels:** We have moved repetitive scatter plots and duplicate metrics to the supplemental figures.
- **Reordered Narrative:** We have re-ordered the figures to follow the new logical flow of the text: moving from clinical validation (Fig. 1-2), to ground-truth experimental benchmarks (Fig. 3-4), and finally to computational prediction (Fig. 5).

It is hard to see the ligands displayed in yellow (Figure 2).

We thank the reviewer for pointing this out. We have updated this figure (now Figure 5) to improve the visibility of the ligands, ensuring they clearly stand out against the protein structure.

Typo in Figure 2: “predicted variant structural stability.”

We thank the reviewer for spotting this error. As part of the figure simplification and reorganization described above (Response to Minor Comment 1), the specific panel containing this text has been removed from the final manuscript, and the typo is therefore no longer present.

Figure 1G should more clearly distinguish the ClinVar variants that are core vs. non-core

We thank the reviewer for this suggestion. We have revised this panel (now Figure 1A) to explicitly state this information.

References:

Cagiada, Matteo, Kristoffer E. Johansson, Audrone Valanciute, Sofie V. Nielsen, Rasmus Hartmann-Petersen, Jun J. Yang, Douglas M. Fowler, Amelie Stein, and Kresten Lindorff-Larsen. 2021. “Understanding the Origins of Loss of Protein Function by Analyzing the Effects of Thousands of Variants on Activity and Abundance.” *Molecular Biology and Evolution* 38 (8): 3235–46.

Findlay, Gregory M., Riza M. Daza, Beth Martin, Melissa D. Zhang, Anh P. Leith, Molly Gasperini, Joseph D. Janizek, Xingfan Huang, Lea M. Starita, and Jay Shendure. 2018. “Accurate Classification of BRCA1 Variants with Saturation Genome Editing.” *Nature* 562 (7726): 217–22.

Livesey, Benjamin J., and Joseph A. Marsh. 2023. “Updated Benchmarking of Variant Effect Predictors Using Deep Mutational Scanning.” *Molecular Systems Biology* 19 (8): e11474.

Olvera-León, Rebeca, Fang Zhang, Victoria Offord, Yajie Zhao, Hong Kee Tan, Prashant Gupta, Tuya Pal, et al. 2024. “High-Resolution Functional Mapping of RAD51C by Saturation Genome Editing.” *Cell* 187 (20): 5719-5734.e19.

Waters, Andrew J., Timothy Brendler-Spaeth, Danielle Smith, Victoria Offord, Hong Kee Tan, Yajie Zhao, Sofia Obolenski, et al. 2024. “Saturation Genome Editing of BAP1 Functionally Classifies Somatic and Germline Variants.” *Nature Genetics* 56 (7): 1434–45.

We thank both reviewers for their constructive feedback and thoughtful suggestions, which have helped improve the quality and clarity of our manuscript. We provide a point-by-point response below.

Reviewer #1 (Remarks to the Author):

This added sentence in the introduction "Most pathogenic LOF variants are missense mutations" is wildly untrue. Please remember that nonsense mediated decay and splicing are a thing and damaging the RNA is epistatic to and potentially even more damaging than missense variation that may be buffered by chaparones in cells. For single nucleotide variants labelled pathogenic or likely pathogenic in ClinVar (179,885), missense (68,859) only slightly outnumber nonsense (67,098), even though missense variants are much easier to make with a single base change. Nonsense (67,098) + splice (38,717) variants greatly outnumber missense variants (68,859). And even then, I'd bet that many of the pathogenic missense variants are actually causing splice defects if you dig into the details. In fact, many clinical geneticists don't even believe missense LOF variants causing protein-effects can be pathogenic at all for some genes (e.g. the new VCEP rules for PALB2 variant classification). Delete this sentence.

Otherwise the updated version has addressed my concerns.
We thank the reviewer for this important correction. We agree the statement was inaccurate and have deleted it from the revised manuscript.

Reviewer #2 (Remarks to the Author):
The paper has been improved significantly from the first submission and my reviews were addressed. I have no additional comments or concerns.
We thank the reviewer for their positive assessment and are pleased that our revisions have addressed their concerns.

(0)

- [x] **Likely pathogenic** (96,486)
- [x] **Pathogenic** (107,707)

Molecular consequence

- [ ] Frameshift (61)
- [ ] Missense (68,859)
- [ ] Nonsense (67,098)
- [ ] Splice site (38,717)
- [ ] ncRNA (19,534)
- [ ] Near gene (0)
- [ ] UTR (7,412)

Variation type clear

- [ ] Deletion (57)
- [ ] Duplication (17)
- [ ] Indel (8)
- [ ] Insertion (32)
- [x] **Single nucleotide** (179,885)
